# An eight-founder wheat MAGIC population allows fine-mapping of flowering time loci and provides novel insights into the genetic control of flowering time

**DOI:** 10.1007/s00122-024-04787-7

**Published:** 2024-11-22

**Authors:** Laure Fourquet, Tobias Barber, Camila Campos-Mantello, Phil Howell, Beata Orman-Ligeza, Lawrence Percival-Alwyn, Gemma A. Rose, Hester Sheehan, Tally I. C. Wright, Friedrich Longin, Tobias Würschum, Dario Novoselovic, Andy J. Greenland, Ian J. Mackay, James Cockram, Alison R. Bentley

**Affiliations:** 1 NIAB, 93 Lawrence Weaver Road, Cambridge, CB3 0LE UK; 2 State Plant Breeding Institute, University of Hohenheim, Hohenheim, Germany; 3 Agricultural Institute Osjek, 31000 Osijek, Croatia; 4 Scotland’s Rural College (SRUC), Kings Buildings, West Mains Road, Edinburgh, EH9 3JG UK

**Keywords:** Multi-founder crop populations, Environmental adaptation, Photoperiod, *Earliness* per se (*Eps*), Flowering time

## Abstract

Flowering time synchronizes reproductive development with favorable environmental conditions to optimize yield. Improved understanding of the genetic control of flowering will help optimize varietal adaptation to future agricultural systems under climate change. Here, we investigate the genetic basis of flowering time in winter wheat (*Triticum aestivum* L.) using an eight-founder multi-parent advanced generation intercross (MAGIC) population. Flowering time data was collected from field trials across six growing seasons in the United Kingdom, followed by genetic analysis using a combination of linear modelling, simple interval mapping and composite interval mapping, using either single markers or founder haplotype probabilities. We detected 57 quantitative trait loci (QTL) across three growth stages linked to flowering time, of which 17 QTL were identified only when the major photoperiod response locus *Ppd-D1* was included as a covariate. Of the 57 loci, ten were identified using all genetic mapping approaches and classified as ‘major’ QTL, including homoeologous loci on chromosomes 1B and 1D, and 4A and 4B. Additional *Earliness *per se flowering time QTL were identified, along with growth stage- and year-specific effects. Furthermore, six of the main-effect QTL were found to interact epistatically with *Ppd-D1*. Finally, we exploited residual heterozygosity in the MAGIC recombinant inbred lines to Mendelize the *Earliness *per se QTL *QFt.niab-5A.03*, which was confirmed to modulate flowering time by at least four days. This work provides detailed understanding of the genetic control of phenological variation within varieties relevant to the north-western European wheat genepool, aiding informed manipulation of flowering time in wheat breeding.

**Supplementary Information:**

The online version contains supplementary material available at 10.1007/s00122-024-04787-7.

## Introduction

In hexaploid bread wheat (*Triticum aestivum* L.) the timing of the phase transition from vegetative to reproductive development, which is correlated with flowering time, is a crucial period for determining final grain yield (Bentley et al. 2013a). Under field conditions, crop life-cycle duration can be manipulated through agronomic means (such as changing sowing date and planting density) or through exploitation of genetic variation for floral transition and development. Genetic studies of flowering time in wheat have often focused on genes with large phenotypic effect, manifesting as developmental responses to the strong environmental cues of daylength (photoperiod response) and low non-freezing temperature (vernalization response). The *Photoperiod-1* (*Ppd-1*) genes confer major control of daylength response (Cockram et al. 2007). Allelic variation at the *Ppd-D1* locus has a major influence on flowering time, with the photoperiod insensitive *Ppd-D1a* allele conferring early flowering under both long-day and short-day photoperiods (Beales et al. 2007; Bentley et al. 2011, 2013b). This locus has been routinely exploited in European winter wheat breeding, with the photoperiod insensitive *Ppd-D1a* allele prevalent in cultivars grown in central and southern Europe (Worland 1996; Bentley et al. 2014), providing a 7–12 day shift in flowering date in both spring (Bentley et al. 2011) and winter wheat (Bentley et al. 2013b).

Although the major genetic controllers of flowering time have been studied, much remains to be discovered about the numerous smaller effect loci, as well as their interaction with major-effect gene variation. A number of smaller effect quantitative trait loci (QTL), termed *Earliness *per se (*Eps*) loci, have been shown to regulate flowering time, with the precise term used to define *Eps* loci variously described (summarized by Cockram et al. 2007; Zikhali and Griffiths 2015). *Eps* QTL have been reported on most wheat chromosomes (e.g. Griffiths et al. 2009). However, relatively few *Eps* loci have been fine-mapped (Valarik et al. 2006; Zikhali and Griffiths 2015; Zikhali et al. 2016; Gawronski and Schnurbusch 2012) and most remain poorly understood, particularly in relation to their interaction with major effect adaptation genes and the environment.

The best characterized wheat *Eps* loci are on the group 1 chromosomes. Initial mapping and characterization of the *Eps* locus *Eps-A*^*m*^*1* in the diploid wheat species *T. monococcum* found it to be thermosensitive (Bullrich et al. 2002), and to affect grain number per spike (Faricelli et al. 2010). Fine-mapping found the *Eps-A*^*m*^*1* genetic interval to contain the candidate gene *EARLY FLOWERING 3* (*EFL3*) (Alvarez et al. 2016). More recently, the orthologous bread wheat *Eps-D1* locus on chromosome 1D was shown to span a region encompassing the candidate genes *EFL3-D1, MODIFIER OF TRANSCRIPTION 1* (*MOT1*) and *FTSH PROTEASE 4* (*FTSH4*) (Zikhali et al. 2016; Wittern et al. 2023). Additionally, the bread wheat *Eps* locus *Eps-B1* has been identified at a broadly homoeologous genomic position to *Eps-A*^*m*^*1* and *Eps-D1* (Zikhali et al. 2016; Wittern et al. 2023), indicating that allelic variation at orthologous genes may control flowering time across wheat sub-genomes and species.

As well as the wheat group-1 chromosome *Eps* loci, *Eps-3A*^*m*^ has been fine-mapped in *T. monococcum* (Gawroński and Schnurbush 2012). It affects flowering and spike characters in a temperature-dependent manner, with mutation resulting in late flowering under short-day and long-day photoperiods (Gawroński et al. 2014). A wheat homologue of *LUX ARRHYTHMO/PHYTOCLOCK 1* (*LUX/PCL1*) has been identified as a candidate gene for *Eps-3A*^*m*^ (Gawroński et al. 2014). In Arabidopsis, LUX/PCL1 is a component of a protein complex termed the Evening Complex, which along with ELF3 and ELF4, acts to regulate the circadian clock (reviewed by Oakenfull and Davis 2017). The wheat line carrying the late flowering allele at *Eps-3A*^*m*^ has a disrupted circadian clock (Gawroński et al. 2014), with *LUX/PCL1* proposed to repress expression of the floral promoter *VRN3* (also known as *FLOWERING LOCUS T, FT*) via negative regulation of *Ppd-1* homoeologue(s) (Mizuno et al. 2012).

Bi-parental mapping populations are commonly used for QTL mapping in wheat but are now complemented by multi-founder populations such as nested association mapping (NAM) and multi-founder advanced generation inter-cross (MAGIC) populations (Ladejobi et al. 2016; Cockram and Mackay 2018; Scott et al. 2020, 2021; Wright et al. 2024). The ‘NIAB Elite MAGIC’ wheat population is one such example (Mackay et al. 2014). Derived from eight recent United Kingdom (UK) cultivars intercrossed over three generations, it is composed of around 1,000 recombinant inbred lines (RILs). It has been demonstrated to be an effective mapping resource for trait dissection and genetic marker development (e.g. Mackay et al. 2014; Cockram et al. 2015; Camargo et al. 2016; Camargo et al. 2018; Downie et al. 2018; Bouvet et al. 2022a, 2022b; Zanella et al. 2023), displaying both high genetic recombination and allelic diversity of relevance to wheat breeding programs in the UK and North-western Europe. Multiple statistical methods are available for MAGIC genetic analyses, including those that correlate phenotype with single nucleotide polymorphisms (SNPs) and those that correlate phenotypes with founder haplotype probabilities via calculation of identity by descent (IBD) coefficients for each genomic location based on marker data, either from the pedigree (Mpmap; Huang and George 2011), or independently of the pedigree (HAPPY; Mott et al. 2000). If the QTL variation is multi-allelic, the IBD approach can offer improved detection due to accounting for multi-founder haplotypes at the locus. Alternatively, if the QTL variation is bi-allelic the IBD approach is less favourable (compared to mapping with SNPs) as more degrees of freedom are required for the test (Milner et al. 2016). Therefore, it is useful to compare results across trial series and methods to determine the accuracy of QTL detection.

In this study, we used an eight-founder MAGIC population to dissect the genetic control of wheat flowering time across a six season field trial data series. Our analyses used both SNP and founder haplotype probability methods to detect genetic loci controlling flowering time. Genetic interactions with the major flowering time gene *Ppd-D1* were assessed to identify additional useful minor locus variation for future exploitation. An additional validation environment was used to further test the environmental robustness of QTL effect in a subset of the MAGIC population. Finally, we exploited residual heterozygosity in the MAGIC lines to Mendelize one of the identified flowering time QTL, *QFt.niab-5A.03*.

## Materials and methods

### Plant material, trial design and field assessments

Details of the ‘NIAB Elite MAGIC’ population are previously described (Mackay et al. 2014; accompanying datasets also available via https://www.niab.com/research/agricultural-crop-research/resources/niab-magic-population-resources). Briefly, the eight founders (UK cultivars ‘Alchemy’, ‘Brompton’, ‘Claire’, ‘Hereward’, ‘Rialto’, ‘Robigus’, ‘Soissons’ and ‘Xi19’) were intercrossed over three generations, whereby all 28 possible two-way combinations were made in the first round of intercrossing, all 210 four-way combinations in the second round, and 210 of the possible 315 eight-way crosses in the third round. Outputs of this crossing scheme were self-pollinated across several generations by single-seed descent to produce > 1,000 RILs, which were genotyped at the F_5_ stage using a 90,000 feature SNP array (Mackay et al. 2014). The only intentional selection made in the population was against extreme dwarf RILs that contain dwarfing alleles at both *RHT-B1* and *RHT-D1* (Gardner et al. 2016), and while ~ 10% of SNPs showed strong segregation distortion, 70% of these were located at three chromosomal positions known to carry introgressions from other species (Gardner et al. 2016).

The population was grown in seven field trials (one trial per year in harvest seasons 2011 and 2013–2016, and two trials in harvest year 2012), located at NIAB, Cambridge, UK (Table 1). At the Cambridge trial sites, daylength on 1st June = 16 h 27 min; additional weather data is recorded in Supplementary Table S1. Each trial was sown in the autumn and reached maturity in the summer of the following calendar year. The year cited in each trial ID name corresponds to the harvest year. The 2011, 2012, 2015 and 2016 ‘nursery’ trials consisted of unreplicated Hege 95B precision drill 1 × 1 m^2^ plots, while the 2012, 2013 and 2014 ‘yield’ trials consisted of partially replicated (p-rep; with one or two replicates of each line arranged in a trial design consisting of blocks and sub-blocks, rows and columns) 6 × 2 m^2^ plots. The growth stages phenotyped were: flag leaf blade all visible (Zadoks growth stage GS39) (Zadoks et al. 1974), half of ear emerged above flag leaf ligule (GS55) and start of flowering (GS61). In the unreplicated 1 × 1 m^2^ plot trials in 2011 and 2012, successive scores of growth stage were measured for all plots on five separate days. In 2012, a second trial consisting of 2 × 6 m^2^ plots arranged in a p-rep design was assessed for development stage on two dates. The total phenotypic data matrix consisted of 16 trait-experiment combinations, as listed in Table 1. 864.

**Table 1:** Summary details of the eight winter-sown field trials undertaken at the NIAB trial site in the United Kingdom (trials 1–7) and at Hohenheim in Germany (trial 8)

Trial	Year	Trial ID	Replication	Trait scored	MAGIC RIL generation	Trial entries	Genotype entries	Plot sizes
1	2011	BLUE_2011	Unreplicated randomised	BLUE over 5 scores on 1–9 scale	F_4_ (F_3_ plants)	1241	642	1 × 1 m^2^
2	2012	BLUE_2012	Unreplicated randomised	BLUE over 5 scores on 1–9 scale	F_5_ (F_4_ plants)	1257	643	1 × 1 m^2^
3	2012	GS_M2012	P-rep design (1 or 2 reps)	GS score on 28th May	F_5_ (F_4_ bulks)	1055	631	2 × 6 m^2^
3	2012	GS_J2012	P-rep design (1 or 2 reps)	GS score on 11th June	F_5_ (F_4_ bulks)	1055	631	2 × 6 m^2^
4	2013	GS39_2013	P-rep design (1 or 2 reps)	Days to GS39	F_6_ (F_5_ plants)	784	639	2 × 6 m^2^
4	2013	GS55_2013	P-rep design (1 or 2 reps)	Days to GS55	F_6_ (F_5_ plants)	784	639	2 × 6 m^2^
4	2013	GS61_2013	P-rep design (1 or 2 reps)	Days to GS61	F_6_ (F_5_ plants)	784	639	2 × 6 m^2^
5	2014	GS39_2014	P-rep design (1 or 2 reps)	Days to GS39	F_7_ (F_6_ plants)	784	639	2 × 6 m^2^
5	2014	GS55_2014	P-rep design (1 or 2 reps)	Days to GS55	F_7_ (F_6_ plants)	784	639	2 × 6 m^2^
5	2014	GS61_2014	P-rep design (1 or 2 reps)	Days to GS61	F_7_ (F_6_ plants)	784	639	2 × 6 m^2^
6	2015	GS39_2015	Unreplicated randomised	Days to GS39	F_8_ (F_7_ plants)	1083	639	1 × 1 m^2^
6	2015	GS55_2015	Unreplicated randomised	Days to GS55	F_8_ (F_7_ plants)	1083	639	1 × 1 m^2^
6	2015	GS61_2015	Unreplicated randomised	Days to GS61	F_8_ (F_7_ plants)	1083	639	1 × 1 m^2^
7	2016	GS39_2016	Unreplicated randomised	Days to GS39	F_9_ (F_8_ plants)	1083	639	1 × 1 m^2^
7	2016	GS55_2016	Unreplicated randomised	Days to GS55	F_9_ (F_8_ plants)	1083	639	1 × 1 m^2^
7	2016	GS61_2016	Unreplicated randomised	Days to GS61	F_9_ (F_8_ plants)	1083	639	1 × 1 m^2^
8	2015	HOH_2015	Unreplicated randomised + repl fitted values	Days to GS55	F_8_ (F_7_ plants)	407	338	2 × 6 m^2^

MAGIC RILs were previously genotyped with the 90 k Illumina iSelect SNP array (Wang et al. 2014), as described in Mackay et al. (2014). Of these, a subset of 643 lines with highly curated SNP genotypic data were used to create a genetic map (Gardner et al. 2016). In this study, the number of MAGIC RILs assessed and with high-quality genotypic data varied by year and by which of the three growth stage assessments were undertaken (summarized in Table 1). However, each trial always comprised the core block of 643 genotyped RILs with the subset of 7,369 high-quality SNP markers anchored in the MAGIC genetic map. To further explore the performance of MAGIC lines outside of the UK environment, GS55 was phenotyped on a subset of 288 genotyped MAGIC RILs in an unreplicated, randomized field trial at Hohenheim, Germany in 2015 (trial HOH_2015) with MAGIC founders replicated for use as fitted values.

### Trial analysis and meta-analysis

As different flowering time scores were used in the 16 trait-experiment combinations (Table 1), it was not possible to use a simple statistical method for analyzing and combining all the data across trials. For the 2011 and 2012 ‘nursery’ trials (1 × 1 m^2^ plots), visual flowering progression scores were scored on two and five days, respectively. For each of these two trials, the progression scores were combined by fitting a linear model with effects for scores and lines to produce best linear unbiased estimates (BLUEs) using the software Genstat 19th edition (VSN International). For the 2013 and 2014 ‘yield’ trials (2 × 6 m^2^ plots), best linear unbiased predictors (BLUPs) were calculated for each line using the ‘lme4’ package (Bates et al. 2015) in R (R Core Team 2017) using the model with the lowest Akaike Information Criterion (AIC) and Bayesian Information Criterion (BIC). The final selected models for the 2013 and 2014 p-rep trials are given in Supplementary Table S2. The trials in 2015 and 2016 were unreplicated, and so no further trials analyses were undertaken. For all 2013–2016 experiments, the date to reach each of the three target growth stages (GS39, GS55 and GS61) were scored in all trials, allowing a meta-analysis across the four years. The BLUPs for each growth stage were calculated for each line with a simple model taking the year as variable using the ‘lme4’ package in R, as Trait ~ Year + (1|line). To account for the different trial designs between years, each line was weighted with 1/variance of the line from the individual trial analyses. For the 2015 and 2016 trials, which only had only a single replicate, the average of the variance of the lines which had one replicate in 2013 and 2014 was used. The BLUPs of each growth stage were then assessed with the same methods as for the single-year traits.

### Effect of *Ppd-D1*

The MAGIC founder cultivar ‘Soissons’ carries the early flowering photoperiod insensitive *Ppd-D1a* mutant allele. To evaluate the effect of *Ppd-D1* on each trait-experiment combination, we genotyped the MAGIC population using a previously described molecular assay (Beales et al. 2007). A linear model was then used to evaluate the effect of *Ppd-D1* on each trait-environment combination: Trait ~ b_0_ + b_1_X_1_ + b_2_X_2,_ where X_1_ = *Ppd-D1* genotype (coded 0, 1 and 2 for number of *Ppd-D1* alleles) and X_2_ = *Ppd-D1* genotype (coded 0 or 2 for homozygotes and 1 for heterozygotes).

### QTL detection

The 16 trait-environment scores, as well as the three growth stage scores from the meta-analysis, were used for QTL analysis undertaken in R. Firstly, each SNP was used in a separate linear mixed model as previously described in Mackay et al. (2014). SNPs were coded 0, 1, 2 (with 1 being the heterozygous class) and included as a fixed regression term, with random effects included to account for relationships among the RILs. This required inclusion of random terms to account for difference between funnel crossing schemes (*funn*) and for differences between outcrossed individuals within funnels from which the RILs were derived (*funn:plant*). The model used for this analysis, denoted ‘SNP’, was Trait ~ (1|funn) + (1|funn:plant) + SNP. In addition, the genotype scores for *Ppd-D1* were included both as a fixed main effect and as an interaction term with the SNP to increase power for detecting main SNP effects and to identify interactions using a model denoted ‘SNP_PPD’ as Trait ~ (1|funn) + (1|funn:plant) + SNP + Ppd-D1 + Ppd-D1:SNP.

Secondly, traits were regressed on the estimated founder haplotype probabilities. This approach was undertaken using the probability of identity by descent (p(IBD)) carried out using ‘mpMap’ in R (Huang and George 2011) with the mpprob function. As with the SNP analysis, regression on the founder haplotype probabilities (via the p(IBD) coefficients) used a mixed model both in the absence (denoted ‘IBD’ as Trait ~ (1|funn) + (1|funn:plant) + p(IBD)) and presence of *Ppd-D1* (denoted ‘IBD_PPD’ as Trait ~ (1|funn) + (1|funn:plant) + p(IBD) + Ppd-D1 + Ppd-D1:p(IBD)). In the case of model overfitting (where residual variance or error = 0), the variable (1|funn:plant) was dropped. To test the significance of main effects, the *q*-value for each marker was calculated. For each combination of trait and analysis method, a *q*-value threshold of 0.05 was estimated from a spline of -log_10_(*p*-value) against -log_10_*p*(*q*-value). For the interaction with *Ppd-D1*, a Bonferroni correction was used with a threshold of 0.05/(number of haplotypes). The number of haplotypes was estimated as 237 based on the length of genetic map (54; Gardner et al. 2016) × four generations of recombination (3 intercrosses + 1/2^n^ at each self-pollinating generation rounded to 1 in total) plus the 21 chromosomes that make up the genetic map. This gives a threshold value of 0.05/237 = 0.00021097 and 3.67 after -log_10_ transformation. The four linear models described above were also run on the additional validation data from trial HOH_2015. The resulting QTL were compared with those found for the main 16 trait-environments and meta-analysis scores for the three growth stages.

In addition to linear modelling, interval mapping was used for the 16 trait-environment combinations and the growth stage meta-analysis scores using ‘mpMap’. Simple interval mapping (SIM) was undertaken with no covariates, as well as composite interval mapping (CIM) with either two (denoted CIM_ncov2) or ten (denoted CIM_ncov10) covariates. Interval mapping significance thresholds were calculated using 100 random simulations.

### QTL definition

To define QTL intervals, the QTL mapping *p*-value outputs were aligned for all trait-environment combinations and analysis methods. These were converted to a -log_10_(*p*-value) and the respective threshold subtracted. QTL were then defined as an interval that contained at least one significant marker based on at least one analysis method for one of the trait-environment combinations. When several markers were significant and genetically close to each other, they were defined as a single QTL. In the situation where two QTL were close to each other and synchronized (significant for the same trait and analysis method, with similar predicted founder effects) they were re-grouped into a single QTL. The QTL peak marker was defined as the marker with the highest significance that was detected for the most trait-environment combinations and across the most analysis methods. Marker order and QTL intervals were confirmed by realigning the genetic markers with the wheat reference genome assembly of cultivar 'Chinese Spring' RefSeq v1.0 (IWGSC, 2018).

### Analysis of QTL physical intervals and gene models

For each QTL, significant SNPs were assigned to recombination bins according to the existing genetic map (Gardner et al. 2016) and all genetically mapped markers within the bin were extracted. These markers were aligned to the wheat reference genome via BLASTn (Altschul et al. 1990) using ~ 100 bp queries with an *e*-value threshold of 1*e*−5. For markers with an equal match on multiple homoeologues/paralogues, physical map position was allocated to the chromosome to which the marker mapped in the genetic map. QTL physical intervals were thus defined by the maximal physical size of the recombination bin(s) within which significant SNPs were located. These were defined by CIM-cov10 or CIM-cov2 when QTL were detected by both IM and CIM; otherwise, intervals were defined by IM or by SNP/IBD results alone. Wheat gene models (RefSeq v1.0) within these physical regions were extracted, along with their gene descriptions, gene ontology (GO) terms and pfam terms, and used for identification of candidate genes based on the available literature. For comparisons of genetic and physical map positions, genetically mapped SNPs for a given chromosome were plotted against physical map position, following the methods described by Gardner et al. (2016). Identification of wheat homologues/orthologues of known rice flowering time genes was undertaken by using the coding regions (CDS) for BLASTn searches of the genome assemblies of different wheat cultivars or species (*T. aestivum* cvs. ‘Chinese Spring’, ‘Claire’, ‘Paragon’, ‘Robigus’ and ‘Synthetic W7984’, *T. monococcum* ssp. *aegilopoides* accession G3116, *T. monococcum* ssp. *monococcum* accession DV92, *T. uratu* accession G1812. *Aegilops tauschii* accession AL8/78) via the GrassRoots webpage: https://wheatis.tgac.ac.uk/grassroots-portal/blast.

### Creation and phenotyping of a near isogenic line for QTL *QFt.niab-5A.03*

A near isogenic line (NIL) for one of the flowering time QTL identified, *QFt.niab-5A.03*, was created via the heterogeneous inbred family (HIF) approach, following the methods described by Zanella et al. (2023). Briefly, using the available 90 k SNP genotypic data, MAGIC RILs heterozygous across the *QFt.niab-5A.03* interval were identified. The likely founders contributing to this heterozygosity were determined by comparing RIL genotypic score at each SNP within the wider region of heterozygosity (i.e. homozygous genotype call A:A, homozygous call B:B or heterozygous call A:B) with the genotype calls at each of the eight founders. Founder contributions at each identified heterozygous locus spanning the QTL were compared to the predicted allelic effects determined in the preceding IM/CIM QTL analyses. This comparison allowed identification of RILs in which the region of heterozygosity at the target locus originated from MAGIC founders with alleles showing strong contrasting effects on flowering time.

Once identified, 29 F_5_ sibling seeds of one such selected MAGIC RIL (MEL_084_2) were grown, and DNA extracted from each individual using a modified Tanksley method (Fulton et al. 1995). A co-dominant Kompetitive Allele-Specific PCR (KASP) marker was developed from the 90 k SNP array marker *Kukri_c14889_116* (known from the RIL genotyping to be heterozygous at the locus) to genotype the F_5_ sibling seed. The KASP primers (VIC-labeled allele-specific primer 5’-cgacagatttcactttgccagaT-3’, FAM-labeled allele-specific primer 5’-cgacagatttcactttgccagac-3’, and common primer 5’-aaaagccagctgatgccagt-3’) were used to genotype the F_5_ sibling individuals, as described by Zanella et al. (2023). Of these, selected individuals with A:A, B:B and A:B KASP genotype scores were grown in compost filled pots under glasshouse conditions to maturity, and the self-pollinated seed from each used to sow single 1 × 1 m^2^ ‘nursery’ plots near Duxford, UK (Latitude 52.08522, Longitude 0.15327) in Autumn 2021, with plants reaching maturity in August of the following year. Additionally, the lines were grown again at a similar location (Latitude 52.10107, Longitude 0.13059) in the 2022–2023 season using 2 × 6 m plots, with six replicates of the early and late allele. The replicates came from three HIF lines carrying the early allele and two HIF lines carrying the late allele. Flowering time was measured every one-to-two days from ear emergence to the start of anthesis using the Zadoks growth scale. Plots were recorded as reaching a specific growth stage when half of the plot had reached the target stage. For the 2022–2023 season trial, a two-sample Welch’s t-test was used to test the allelic effect on flowering time between the replicates, using the software R. The Welch’s t-test was used due to the unequal variances of the two allele groups being compared. A boxplot showing the variation was formed using the R packages *ggplot2* (Wickham 2016) and *ggsignif* (Ahlmann-Eltze and Patil 2021).

## Results

### Field assessments and phenotypic analysis

**Fig. 1:**
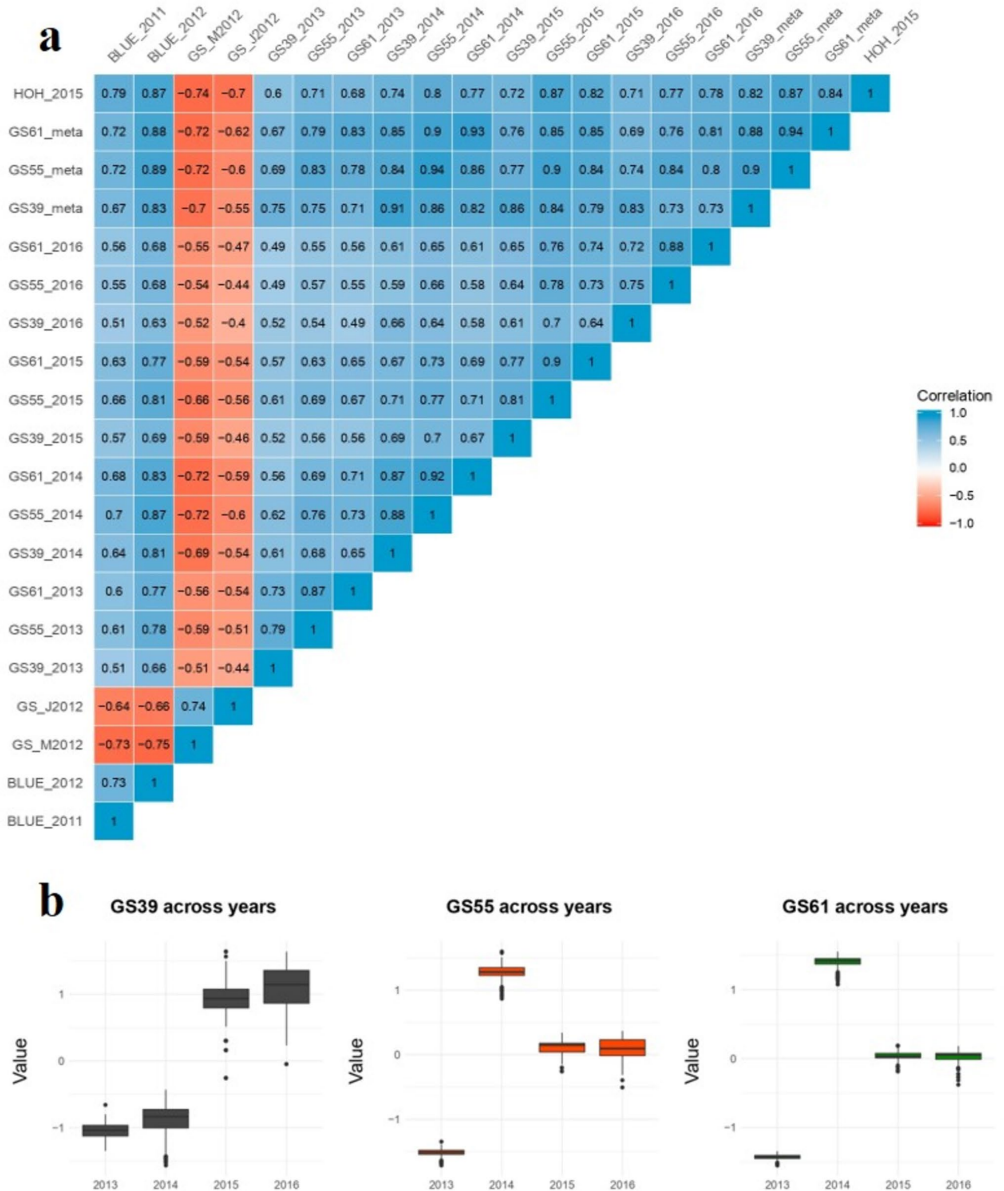
Flowering time data analysis. **a** Correlations among the 16 UK trait-environment combinations, the growth stage meta-analysis and the HOH_2015 datasets. GS39 = flag leaf blade all visible, GS55 = half of ear emerged above flag leaf ligule, GS61 = start of flowering. **b** Boxplots of MAGIC RIL phenotypic scores in the 2013, 2014, 2015 and 2016 season trials for growth stage GS39, GS55 and GS61

A matrix of correlations among the 16 UK trait-environment combinations, the growth stage meta-analysis and HOH_2015 data, showed that the scores were relatively highly correlated (ranging from 0.40–0.94; Fig. 1a). The HOH_2015 validation scores correlated highly with UK scores, with a maximum of r = 0.87 (between BLUE_2012 and HOH_2015). Two traits, GS_J2012 and GS_M2012, showed a negative correlation with the other scores as their scoring was inverted with high values representing early development. The phenotypes of the 643 lines showed variation between years and the amplitude of variation varied from year to year and was growth stage-dependent (Fig. 1b). *Ppd-D1* was found to have a statistically significant effect on phenotype in all trait-environment combinations (*p*-value < 10^–10^). The proportion of the phenotypic variation accounted for (r^2^) is summarized in Table 2 and ranged from 0.113 (GS39_2013) to 0.546 (GS61_2014). The meta-analysis combined four years of trial data for each of the three growth stages. The year effect was very high, with r^2^ values of 0.997 for GS39 and GS55 and 0.594 for GS61.

**Table 2:** *Ppd-D1* allele effect (r^2^) on the trait-environment datasets analysed

Trait	r^2^ value	*p*-value additive effect
BLUE_2011	0.324	> 10E^−13^
BLUE_2012	0.353	> 10E^−13^
GS_M2012	0.320	> 10E^−13^
GS_J2012	0.139	> 10E^−13^
GS39_2013	0.113	> 10E^−13^
GS55_2013	0.167	> 10E^−13^
GS61_2013	0.176	> 10E^−13^
GS39_2014	0.487	> 10E^−13^
GS55_2014	0.423	> 10E^−13^
GS61_2014	0.546	> 10E^−13^
GS39_2015	0.325	> 10E^−13^
GS55_2015	0.278	> 10E^−13^
GS61_2015	0.260	> 10E^−13^
GS39_2016	0.217	> 10E^−13^
GS55_2016	0.158	> 10E^−13^
GS61_2016	0.195	> 10E^−13^
GS39_meta	0.404	> 10E^−13^
GS55_meta	0.340	> 10E^−13^
GS61_meta	0.432	> 10E^−13^
HOH_2015	0.204	> 10E^−13^

### QTL detection

For main effects, significance thresholds determined as the -log_10_(*p*) values corresponding to a 5% *q*-value threshold ranged from 2.40 to 8.45, with an average of 3.05 for the four linear model analyses on the 16 UK trait-environment scores. For the SIM and CIM analyses, the significance thresholds calculated from 100 simulations ranged from 4.09 to 4.62 with an average of 4.36 (Supplementary Table S3). In total, 57 QTL were detected across all traits and analyses, located on 18 of the 21 wheat chromosomes (Supplementary Tables S4 and S5). Of these QTL, 9 were identified using all genetic analysis methods applied. In addition, a QTL assumed to be conferred by *Ppd-D1* on chromosome 2D (with the *Ppd-D1* gene marker as the most significant marker at this QTL), was identified using all genetic analyses methods, except for SNP_PPD and IBD_PPD in which *Ppd-D1* was used as a covariate. Together, these 10 QTL are denoted as ‘major QTL’ (Table 3 and Supplementary Tables S6 and S7). The significance values and proportion of variation explained by the remaining 47 QTL for each trait where a significant effect was detected is given in Supplementary Table S8.

**Table 3:** Summary details of the 10 ‘major’ quantitative trait loci (QTL) identified across the 16 trait-environment combinations analysed

		Peak SNP			Frequency		Trait-env %var^a^		Meta %var^a^	
QTL ID	Chr^b^	SNP name	cM^c^	Mbp^d^	Trait-env	Meta	Min	Max	Min	Max
*QFt.niab.1B.05*	1B	IAAV5516	349.6	688.710	16	3	0.14	55.81	0.74	45.83
*QFt.niab.1D.03*	1D	Kukri_c29687_369	123.2	493.212	12	3	1.66	50.01	2.13	45.20
*QFt.niab.2D.01*	2D	Ppd_D1	60.7	36.934	16	3	3.20	55.49	29.96	44.89
*QFt.niab.3A.01*	3A	IAAV4781	98.0	107.739	15	3	0.98	54.38	1.38	44.29
*QFt.niab.4A.03*	4A	BS00064140_51	153.2	666.111	16	3	1.51	53.80	1.63	44.89
*QFt.niab.4B.01*	4B	Excalibur_c22632_576	165.6	609.413	13	3	1.27	53.66	2.10	43.68
*QFt.niab.4D.01*	4D	RAC875_c1673_663	32.2	16.587	5	1	0.57	51.19	1.90	43.40
*QFt.niab.6A.03*	6A	Ku_c69999_111	172.4	552.550	12	3	1.40	42.34	1.71	43.48
*QFt.niab.6D.01*	6D	IAAV8527	117.7	389.607	13	3	1.84	54.63	1.80	43.64
*QFt.niab.7B.01*	7B	Excalibur_c29698_76	27.0	15.307	9	2	2.26	53.91	2.00	45.36

^a^Indicated is the minimum (Min) and maximum (Max) percentage of the variation explained in the individual trait-environment analyses (Trait-env %var), as well as in the meta analyses (Meta %var)

^b^Chr = chromosome

^c^Genetic map position as reported byGardner et al. (2016)

^d^Physical map position according to the wheat reference genome of cv. ‘‘Chinese Spring’ (RefSeq v1.0. IWGSC, 2018)

**Fig. 2:**
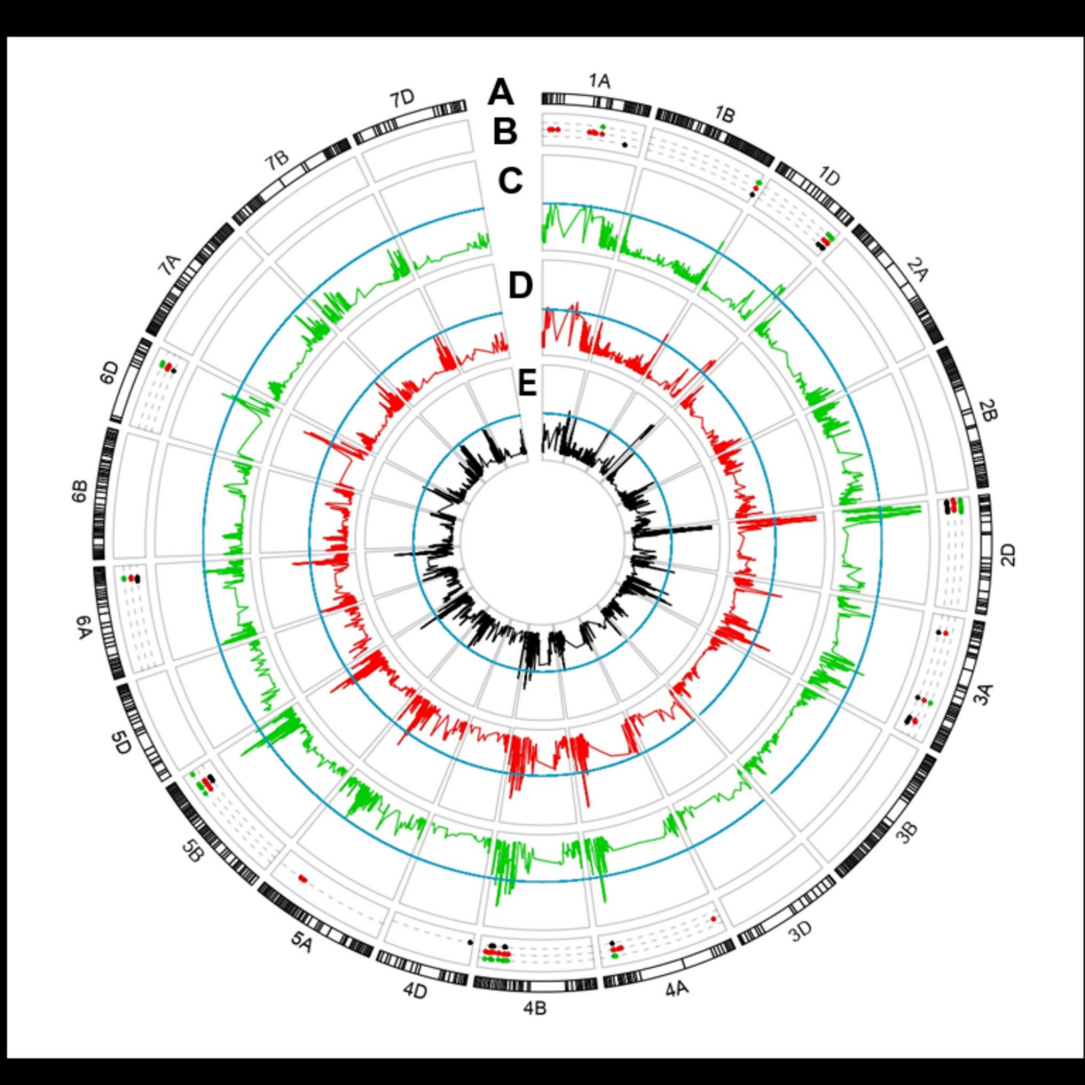
Summary of the QTL identified for growth stages GS39 (half of ear emerged above flag leaf ligule), GS55 (flag leaf blade all visible) and GS61 (start of flowering) using the meta-analysis scores. **a** The 21 chromosomes of wheat, indicating physical map position of SNPs (based on the wheat reference genome assembly, RefSeq v1.0). **b** Locations of significant markers for QTL detected at growth stages GS39 (green), GS55 (red) and GS61 (black). QTL scans for (**c**) GS39_meta, **d** GS55_Meta, and **e** GS61_Meta. In tracks C-E, the significance threshold is shown in blue

Of the 57 QTL detected, 23 were growth stage specific, with more GS-specific QTL identified for GS55 (12) than for GS61 (7) or GS39 (4). A summary of the QTL detected in the meta-analysis scores at GS39 (26 QTL), GS55 (37) and GS61 (32) is presented in Fig. 2. Across years, only three of the 10 major QTL were detected in all trait-environment combinations (*QFt.niab-1B.05*, *QFt.niab-2D.01*, *QFt.niab-4A.03*) whilst 17 of the minor QTL were detected in only one year (Supplementary Table S8). The number of QTL detected in each year also varied, with the lowest number found in 2011 (15 QTL) and the highest in 2014 (29 QTL). A full list of QTL detected in each year (although not necessarily at all growth stages in each year) is given in Supplementary Table S9.

Of the analysis methods tested, the SNP method detected the most QTL (36), of which 10 were not detected by any other methods. IBD_PPD detected 33 QTL while SNP_PPD detected 31 QTL. Method SIM_cov0 detected the least QTL (11), all of which were identified by at least two other methods. IBD detected 17 QTL whilst the CIM_cov10 analysis detected 19 QTL. In total, 20 QTL were only detected by at least one of the methods that used one or several covariates (SNP_PPD, IBD_PPD, CIM_cov2 and/or CIM_cov10). A comparison of QTL detection from the SNP, IBD and CIM_cov10 analysis based on the meta-analysis scores for GS55 is presented in Supplementary Fig. S1.

**Fig. 3:**
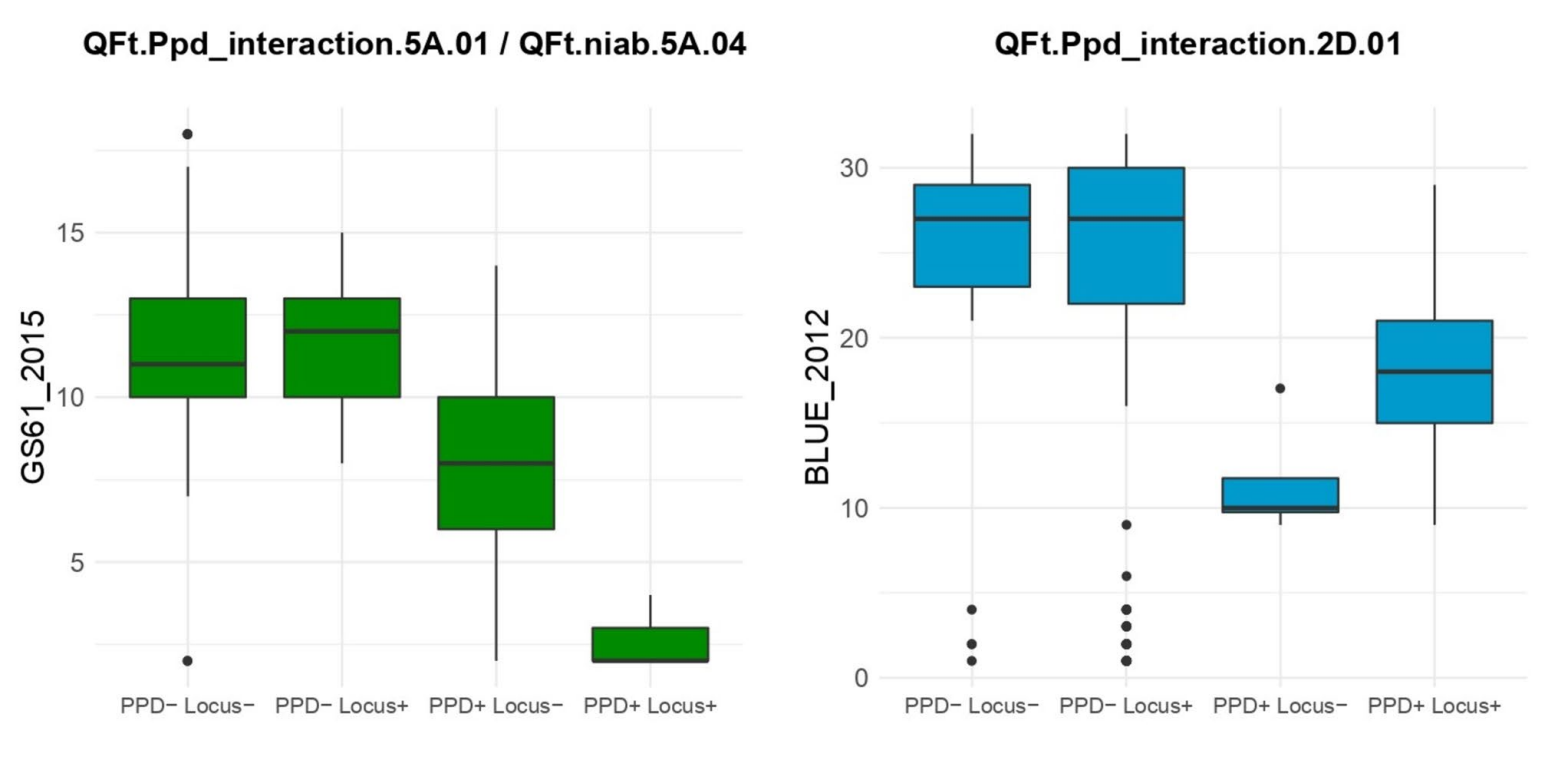
Two examples of the effects on flowering time of genetic loci found to interact with the major flowering time locus *Ppd-D1*. Indicated are the name of the interacting QTL (which includes chromosome designation) magnitude (effect on days to growth stage GS61) and direction of these interactions. PPD- = late flowering *Ppd-D1b* allele, PPD +  = early flowering *Ppd-D1a* allele. Locus- = interacting locus allele 1, Locus +  = interacting locus allele 2. The trait-environment dataset in which the examples shown were identified are indicated on the y-axis. Where interacting loci correspond to main effect QTL, both the interacting name and equivalent main effect name is listed above the boxplot. In both examples, the interacting locus influenced days-to-GS61 only in the presence of the photoperiod insensitive early flowering *Ppd-D1a* allele

The significance of the interactions with *Ppd-D1* were assessed using the linear models SNP_PPD and IBD_PPD for all 16 UK trait-environment and the three growth stage meta-analysis scores. The significance threshold obtained using a haplotype Bonferroni correction was -log_10_(*p*) = 3.67. In total, 31 interaction QTL were detected, of which 12 were detected with both models, 13 by only SNP_PPD, and six with only IBD_PPD. Of these, 11 were in the same interval as main effects QTL (Supplementary Table S10). The nature, direction and magnitude of the *Ppd-D1* interactions varied, with a subset of interactions shown in Fig. 3.

The four linear models (SNP, SNP_PPD, IBD and IBD_PPD) were also run on the additional HOH_2015 data set. The significance thresholds were set to a 5% *q*-value range from 2.70 to 3.32 for the four linear models analyzed (Supplementary Table S3). In total, 13 QTL were detected in the HOH_2015 dataset across the four analysis methods, 11 of which had been detected in the main UK data set for at least one growth stage trait, including five of the ten most important QTL (Supplementary Table S11). Two unique QTL were detected on chromosome 3B (in the IBD_PPD analysis) and 6B (in the IBD and IBD_PPD analyses).

### QTL physical interval and candidate gene analysis

For each QTL, physical intervals and gene content were determined using the wheat reference genome assembly (RefSeq v1.0) and gene model annotations (RefSeq v1.0) (IWGSC, 2018) (Supplementary Table S12). QTL physical intervals ranged from 11.1 Mb (*QFt.niab-1B.05*) to 495.8 Mb (*QFt.niab-6A.03*, which spanned the pericentromeric region of chromosome 6A), with a median QTL size of 98.4 Mb (*QFt.niab-4A.03*). Analysis of the gene content within each QTL interval allowed candidate genes to be identified (Supplementary Table S12).

The interval for *QFt.niab-1B.05* contained 185 gene models, including 11 candidate genes such as *EARLY FLOWERING 3* (*EFL3*)*,* three *CONSTANS*-like genes found here to belong to the Clade 4 *CCT MOTIF FAMILY* (*CMF*) genes (Cockram et al. 2012), and a zinc finger CCCH domain gene. The physical interval for *QFt.niab-1D.03*, located at a homoeologous position on chromosome 1D, was larger (34.7 Mb, 1,471 genes) and contained 45 candidate genes. This included homoeologues of all candidate genes identified for *QFt.niab-1B.05*, except for one of the tandemly repeated *CONSTANS*-like genes and the *APETALA 3*-like MADS-box transcription factor. *QFt.niab-3A.01* spanned the pericentromeric region and so the relatively small genetic interval corresponded to a large physical interval covering more than half of chromosome 3A (67.3—507.3 Mb). This interval contained over 4,500 gene models and ≥ 19 candidate genes. QTL *QFt.niab-4A.03* on chromosome 4A spanned a physical interval of 73.7 Mb, with 13 candidate genes identified from the 1,963 gene models within the interval. Notably, QTL *QFt.niab-4B.01* was located at a homoeologous location on the long arm of chromosome 4B. It had a physical interval of 139.1 Mb containing 2,523 gene models and 22 candidates including *TERMINAL FLOWER 1*-*like*, *FRIGIDA-like* genes, and eight MADS-box genes. The *QFt.niab-4D.01* interval contained 1,144 genes, including six candidate genes, with two genes at the peak of the QTL: a Clade II *COL* gene orthologous to rice gene *OsJ* and a gene annotated as having homology to the Arabidopsis gene, *Protein UPSTREAM OF FLC* (*UFC*). *QFt.niab-6A.03* has a diffuse genetic and physical chromosomal region of significance, spanning > 6,900 genes with nine candidate genes at the QTL peak, including an *FT*-like gene and two Clade I *CO*-like genes. The *QFt.niab-6D.01* physical interval contained 169 genes, including 16 candidate genes, with three *CO-*like genes from Clade I, II and III, eight MADS-box genes, *Phytochrome A* and a *FRIGIDA interacting protein 1*-like gene. Finally, *QFt.niab-7B.01* on chromosome 7B was delimited by a 22.6 Mb interval containing 1,342 gene models and 10 candidate genes: nine MADS-box genes and *FLOWERING LOCUS T-B1* (*FT-B1*), which has been previously identified as playing a central role in the control of flowering time (Yan et al. 2006).

### Mendelization of flowering time QTL, *QFt.niab-5A.03*

**Fig. 4:**
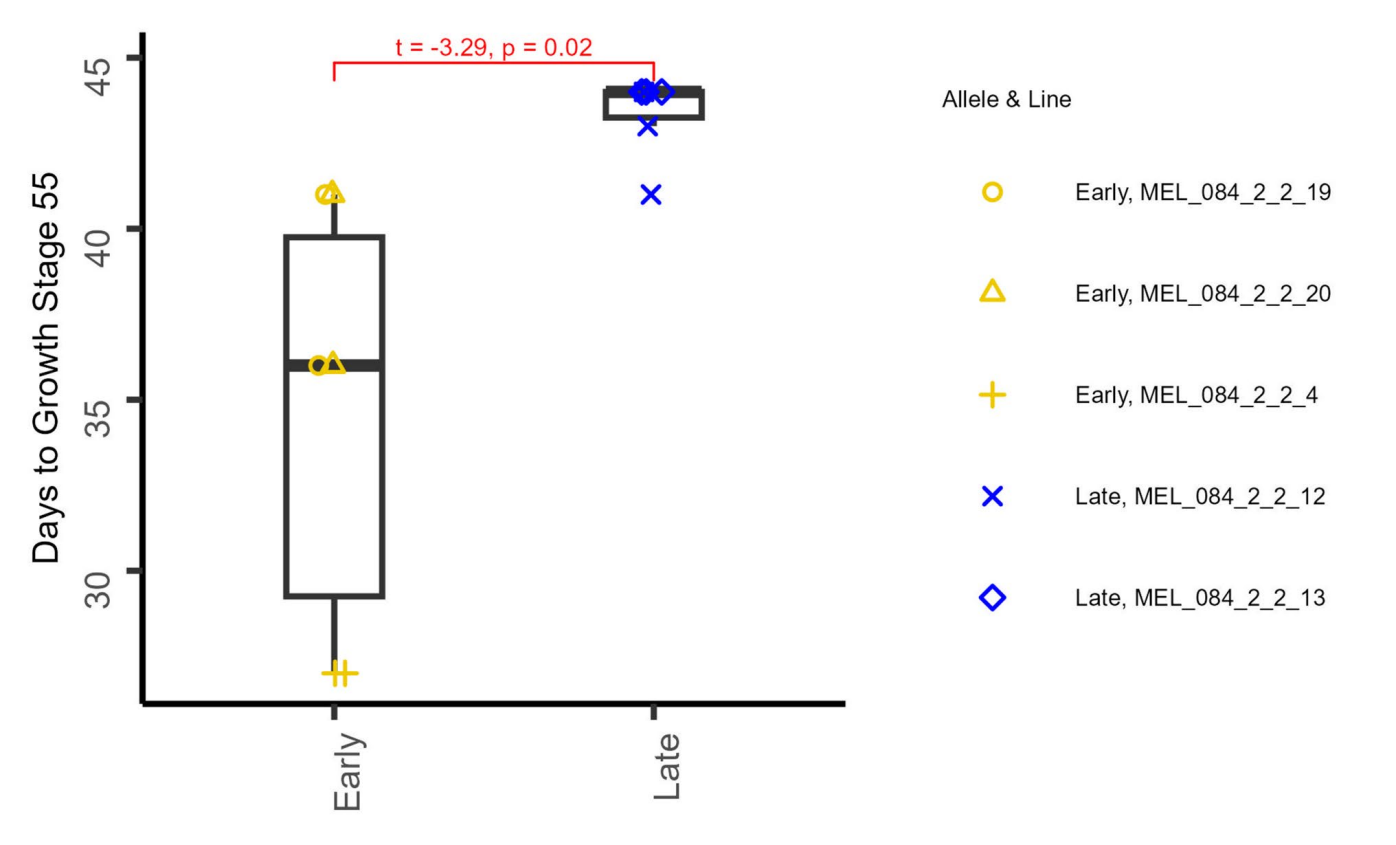
Boxplot of the number of days to reach Zadoks growth stage 55 (GS55, half of ear emerged above flag leaf ligule) of the heterogeneous inbred family (HIF) lines generated for flowering time genetic locus *QFt.niab-5A.03* assessed at the Duxford site in summer 2023. Six plots were assessed per allele, with three lines carrying the early allele and two lines carrying the late allele. Significance between the alleles for days to reach GS55 was assessed via a two-sample Welch’s t-test

We selected ‘minor’ QTL *QFt.niab-5A.03* to develop a nearly isogenic line pair via exploitation of the residual heterozygosity present in the MAGIC RILs. Of the eight RILs identified as being heterozygous across the QTL using the 90 k SNP data, one was selected for forward analysis: MEL_084_2. Heterozygosity at the *QFt.niab-5A.03* locus in this RIL was determined to be due to alleles from the founders ‘Hereward’ and ‘Soissons’, which our QTL analyses predicted to differ in their effect on flowering time by approximately two days. Twenty-nine F_5_ sibling seed of the individual previously genotyped using the 90 k array were grown and genotyped using a KASP marker at the target QTL, with the resulting ratio of observed homozygous and heterozygous individuals (7 A:A, 14 A:B, and 8 B:B) agreeing with the expected 1:2:1 ratio ($\chi$ ^2^ = 1.03, *p*-value = 0.95). Phenotyping 10 of these lines under field conditions found plots homozygous for the late flowering ‘Hereward’ allele to reach 50% ear emergence 4–5 days later than the plots homozygous for the earlier flowering ‘Soissons’ allele (Supplementary Table S12), and in agreement with the direction of allelic effects predicted to be conferred by these alleles in our QTL analysis of the MAGIC population. In the following year, flowering time was measured on six replicates homozygous for each of the ‘Soissons’ and the ‘Hereward’ allele, which originated from three of the early flowering and two of the late flowering HIF lines. The effect of allele was significant (*t* = −3.29, *p*-value = 0.02, Fig. 4), where the replicates with the ‘Soissons’ allele reached ear emergence (GS55) on average nine days earlier than those with the ‘Hereward’ allele. The significance of the line effect corresponding to the different HIF lines was not assessed. However, it was noticeable that in both trials MEL_084_2_2_4 reached GS55 at an earlier date compared to the other HIF lines that also carried the ‘Soissons’ allele (Fig. 4, Supplementary Table S13).

## Discussion

### QTL mapping methods

We used seven analysis methods to detect QTL in 16 flowering-related trait-environment scores collected in the field across six UK growing seasons. The use of multiple analyses revealed notable disparity in the number of QTL detected: from 36 with the SNP method to 11 using SIM. Twenty-three QTL were detected by one method alone, demonstrating the benefit of using multiple mapping methods to define the QTL space. For linear models, the difference in QTL number detected between the simple and covariate models, and between using single markers (SNP) and founder haplotype probabilities (IBD), was large. Including the major flowering time gene *Ppd-D1* allowed identification of additional loci (15 with IBD versus 5 with SNPs). For IBD, all but three QTL detected without *Ppd-D1* as a covariate were also detected in IBD_PPD: *QFt.niab-2D.01* (the QTL spanning *Ppd-D1* itself), *QFt.niab-1A.01* and *QFt.niab-7A.01*. For the SNP analysis, only 19 of the 31 QTL were detected in both SNP and SNP_PPD analyses. Using *Ppd-D1* as a covariate, 12 additional QTL were detected whilst 17 were lost. This highlights the impact of *Ppd-D1* for genetic analyses of flowering time and the importance of accounting for major gene variation in QTL detection, as previously reported in other cereal species, including wheat (Bentley et al. 2014; Guedira et al. 2016; Arjona et al. 2018).

For the three interval mapping methods used (SIM, CIM_cov2 and CIM_cov10), increasing the number of covariates increased the number of QTL detected. It also produced nested results: all QTL detected using SIM were also detected using CIM_cov2 (with the exception of the minor QTL *QFt.niab-5B.03*), and all CIM_cov2 QTL were detected using CIM_cov10. Including covariates in the interval mapping reduced background noise and provided clearer and higher significance peaks with smaller QTL intervals, as previously reported (e.g. Zeng 1994). This meant that some QTL that did not reach the significance thresholds using SIM were significant using CIM_cov2 and CIM_cov10. Overall, CIM methods gave the clearest peaks and the highest confidence QTL but required the most computational time. These results demonstrate that multiple analysis methods should be used for the accurate detection of significant loci in MAGIC, and that simple methods (e.g. using only SNPs) are the least stringent. Furthermore, mapping with multi-allelic founder haplotype probabilities (IBD) can reveal QTL that are not detectable with a bi-allelic single marker approach (e.g. Zanella et al. 2023). In this analysis, several trait/year combinations were only identified using IBD and/or IBD_PPD analyses (Supplementary Table S5). A notable feature of the genetic mapping was variation in QTL interval size. This was predominantly driven by the large physical intervals observed for the nine QTL that spanned or overlapped chromosomal regions with extremely low genetic recombination—such as the pericentromeric regions of each chromosome, and chromosomal introgressions originating from other grass species (such as the wheat/rye chromosome 1B/1R introgression) (Supplementary Table S4). For some of the weaker QTL, genetic loci were identified via single genetic markers, and so the genetic intervals provided must be considered accordingly. For all remaining genetic loci, mean physical QTL interval was 37.3 Mb, indicating reasonably good genetic/physical map resolution. While alternative methods to identify and/or to define QTL intervals may be available, we have explored a variety of genetic analysis methods providing extensive information on the genetic architecture of flowering time in UK wheat across multiple UK trials and seasons. The additional validation field trial in Germany (HOH_2015) confirmed 10 of the QTL detected in the UK, including six of the ten ‘major’ QTL (including *Ppd-D1*) and four of the ‘minor’ QTL. This is a preliminary indication that QTL detected in the UK (using UK wheat germplasm) are likely to be useful sources of flowering time variation in additional European wheat production environments. Additionally, using a subset of the MAGIC population (288 RILs) for field screening of flowering time is useful for detecting at least a portion of the QTL controlling this trait.

### QTL across growth stages

Some QTL were detected differentially across the growth stages, indicating growth stage dependency of the phenotypic effects of these loci. Previous work has identified differences in temporal QTL detection for other traits in the same MAGIC population (Camargo et al. 2018). Understanding the growth stage-specific nature of the genetic architecture underpinning target trait(s) will inform their use in breeding and identification of the underlying genes and causative variants. The highest number of QTL were detected for GS55, the primary selection target for flowering time adaptation, with 12 of these being unique to this growth stage. Across successive growth stages, more QTL were shared between GS55 and GS61 than between GS39 and GS55, which is to be expected as GS55 and GS61 have been previously shown to be highly correlated (e.g. Bentley et al. 2013b). QTL in common for GS39 and GS61 were not expected as there is not a clear biological rationale for loss of effect at the intervening GS55. While two minor QTL (*QFt.niab-2A.01* and *QFt.niab-7B.05*) were detected for this combination, they are relatively weak QTL, indicating that effect at the intervening growth stage could have been missed due to lack of power for robust detection.

*Ppd-D1* is a well-known modulator of wheat flowering time (reviewed by Bentley et al. 2013a). The photoperiod insensitive *Ppd-D1a* allele, present in MAGIC parent ‘Soissons’, accelerates flowering time by approximately 10 days compared to the wild-type *Ppd-D1b* allele in wheat (Bentley et al. 2011; 2013b). We found the QTL at *Ppd-D1* to be highly significant in all analyses and for all growth stages in all years (except for SNP_PPD and IBD_PPD) and explained approximately 30% of the variance. Including the *Ppd-D1* perfect marker as a covariate (SNP_PPD, IBD_PPD) identified additional QTL which could be useful sources of genetic variation for research and breeding, and are explored in more detail below. The SNP_PPD and IBD_PPD models were also used to detect QTL that interacted with *Ppd-D1,* finding 31 interaction QTLs. Of these, 11 coincided with main effect QTL, indicating they are more likely to be involved in the immediate genetic pathway in which *Ppd-D1* operates. This is of interest, as while much research has focused on the effect of *Ppd-D1* and the major vernalization response locus *VRN-1* (e.g. Kumar et al. 2012) on flowering, comparatively little is known about the nature of its molecular interactions with other members of the broader flowering time pathway. For example, genetic interaction between *Ppd-D1* and the flowering time QTL *QFt.niab-1D.03* and *QFt.niab-7B.01* indicates potential close molecular interaction between *PPD-D1* and members of the Evening Complex (*ELF3-D* and *FT1-B*), which integrates molecular signals from the photoperiod and temperature response pathways. PPD-D1 carries a CCT domain towards the C-terminus. As CCT domain proteins are known to interact with both the Evening Complex (e.g., TIMING OF CAB EXPRESSION 1 (TOC1) in Arabidopsis; Huang et al. 2016) and with the Florigen Activation Complex (which contains FT) (e.g., DHD4 in rice; Cai et al. 2021), it would be of interest to explore whether PPD-D1 directly interacts with the proteins that form either of these two complexes. The recent finding by Li et al. (2024) that genetic interaction between photoperiod and floral pathway gene *GIGANTEA* (*GI*) occurs only in the presence of the photoperiod sensitive *Ppd-D1b* allele provides further evidence of the potential importance of direct or indirect interactions between floral pathway genes and PPD-D1.

### Major flowering time loci

Excluding the well characterized *Ppd-D1* locus, the remaining nine ‘major’ QTL identified provide robust sources of genetic variation for breeders to develop cultivars with specific flowering times, enabling targeted adaptation to specific environments. Two QTL were detected on the group-1 chromosomes (*QFt.niab-1B.05; QFt.niab-1D.03*) and correspond to the best characterized wheat *Eps* QTL: *Eps-A*^*m*^*1* (from *T. monococcum*) and *Eps-B1* and *Eps-D1* (*T. aestivum*). *ELF3* has been identified as a candidate for *Eps-A*^*m*^*1* (Alvarez et al. 2016), *Eps-B1*, and *Eps-D1* (Wittern et al. 2023; Zikhali et al. 2016; Lisker et al. 2022), with *MOT1* and *FTSH4* also highlighted as potential candidates. Rice possesses two closely related *ELF3*-like genes, *OsELF3-1* (*LOC_Os06g05060*) and *OsEF3-2* (*LOC_Os01g38530*) (Zhao et al. 2012). Of these, *OsELF3-1* plays a more prominent role in the control heading date in rice (Zhao et al. 2012) and is thought to encode the flowering time QTL *Hd17* (Matsubara et al. 2012).

Loss of function alleles at *ELF3-A* and *ELF3-B* in tetraploid wheat (*T. durum*) have been shown to severely disrupt circadian rhythms (Wittern et al. 2023). Similarly, an artificial mutation in barley *HvELF3* has been found to underly the *early maturity 8* (*eam8*) allele that accelerates transition from vegetative to reproductive growth and inflorescence development (Faure et al. 2012). Despite the pronounced circadian clock defect caused by the mutation, *eam8* has been used in breeding programs to facilitate short growth-season adaptation and expansion of the geographic range of barley cultivation (Faure et al. 2012). Here, we show that alleles at *Eps-B1* (*QFt.niab-1B.05*) and *Eps-D1* (*QFt.niab-1D.03*) segregate in the eight wheat varieties used to create the MAGIC RILs, and that they have a consistent and relatively strong phenotypic effect on flowering. Recent studies in tetraploid wheat show that the ELF3 protein physically interacts with the *Ppd-A1a* promoter (Alvarez et al. 2023), suggesting a likely molecular mechanism underpinning the genetic interaction we observe between the *Ppd-D1* locus and the chromosome 1A and 1B QTLs spanning *ELF3* in our hexaploid wheat MAGIC population. While *ELF3* homoeologues likely remain the best candidate genes for the chromosome 1B and 1D QTL, additional potential candidates are present (see Supplementary Text S1).

Of the two ‘major’ QTL *QFt.niab-4A.03* and *QFt.niab-4B.01* located at broadly homoeologous positions on chromosome 4A and 4B, the shorter *QFt.niab-4A.03* physical interval was completely contained within that of *QFt.niab-4B.01*. Both intervals contained a strong candidate gene: the PEBP domain gene *TERMINAL FLOWER 1-LIKE* (*TFL1-like*). In Arabidopsis, *TFL1* is expressed in the shoot meristem and acts to delay flowering (Shannon and Meeks-Wagner 1991), counteracting the promotion of flowering by *FT*, and helps control final plant architecture via regulation of shoot and floral meristems (Baumannn et al. 2015). Rice has four reported *TFL1* homologs, *RICE CENTRORADIALIS1* (*RCN1*), *RCN2*, *RCN3* and *RCN4*, with *RCN1* and *RCN2* known to delay transition to the reproductive phase (Nakagawa et al. 2002), analogous to the role of *TFL1* in Arabidopsis. The barley *Eps2* locus on chromosome 2H is encoded by the *OsRCN4* orthologue *HvCEN*, allelic variation at which helps adapt barley to contrasting European environmental zones (Comadran et al. 2012). While the homoeologous *TFL1-*like genes at the group-4 MAGIC QTL identified here are not orthologous to the previously characterized rice or barley *TFL1*-like genes, the central role of this class of gene in the modulation of shoot development and flowering indicates they nevertheless represent strong candidates for *QFt.niab-4A.03* and *QFt.niab-4B.01.* Additional candidate genes at these loci are discussed in Supplementary Text S1.

For the *QFt.niab-7B.01* locus, early flowering alleles from founders ‘Brompton’ and ‘Soissons’ were predicted to confer a 1–2 day acceleration in heading date. Of the 10 candidate genes at the locus, nine were MADS-box genes, and the remaining was *FT-B1. FT* genes play a central role in the control of flowering in monocot and dicot species (reviewed by Pin and Nillson 2012). In cereals, Clade I *FT1-*like genes (Haliwell et al. 2016) encode *Hd3a* in rice (Kojima et al. 2002) and the *VRN3* vernalization requirement loci in barley and wheat (Yan et al. 2006). The *VRN3* allele from the wheat cultivar ‘Hope’, which confers extremely early flowering under LDs, originated from an interspecific cross and is only very rarely used in bread wheat cultivars. The effect of the ‘Hope’ allele is dependent on genetic background, resulting in a spring growth habit in an otherwise winter wheat background (Yan et al. 2006; Nichter et al. 2014) or causing more subtle changes in heading date of ~ 2–4 days in backgrounds containing different alleles at the homoeologous *Vrn-1* loci (Nichter et al. 2014). Association mapping approaches have identified the genomic region containing *FT-A1* and/or *FT-B1* to be associated with flowering time and yield in European winter wheat (e.g. Bentley et al. 2014) and in landraces from diverse geographic origins (Bonnin et al. 2008). These results are supported by studies showing that the lines carrying spontaneous chromosomal deletions encompassing *FT-B1* exhibit delayed flowering and altered development in response to ambient temperatures (Dixon et al. 2018; Finnegan et al. 2018). Artificial mutation of *TaFT* in tetraploid wheat (*T. durum*, AABB genome) has shown that a synonymous mutation of *TaFT-B* within the conserved PEBP domain results in late flowering. The *TaFT-B* mutant shows more pronounced delay in flowering than a *TaFT-A* mutant carrying a premature stop codon eliminating half of the predicted protein (Lv et al. 2014). Collectively, investigations of natural and artificial mutations indicate that *TaFT* homoeologues play a central role in the genetic pathways controlling wheat flowering time, supporting *TaFT-B* as a candidate for *QFt.niab-7B.01*. Genetic intervals for the four ‘major’ QTL *QFt.niab-3A.01, QFt.niab-4D.01, QFt.niab-6A.03* and *QFt.niab-6D.01* all spanned regions of extremely low genetic recombination around the pericentromeric regions. As these encompass very large physical intervals, further discussion of gene content is not undertaken here.

### Mendelization of flowering time locus *QFt.niab-5A.03*

Mendelization of QTL by the creation of NILs allows precise investigation of QTL effects in the absence of confounding effects from variation at additional genetic loci controlling the target trait, and is commonly a critical step towards subsequent QTL cloning. By exploiting residual heterozygosity in the MAGIC RILs, we generated a NIL pair for one of the ‘minor’ flowering time QTL identified within the population within just two generations and generated phenotypic data that supported the successful Mendelization of the locus. Such resources will provide the basis for detailed downstream physiological and molecular genetic analyses, helping to further understand the genetic control of flowering time in wheat and support the development of diagnostic genetic markers to track specific alleles in breeding programs.

### Concluding remarks

Wheat is cultivated globally across a wide range of environments both in highly productive agricultural systems and in subsistence agriculture. As the climate changes there is an urgent need to identify varieties adapted to target environment that will consistently yield highly under increased environmental stresses. Manipulating flowering time, to synchronize reproductive development with environmental conditions and optimize grain yield in target environments, is key to this process. Here, we used a multi-parent population to determine a large proportion of the genetic loci controlling flowering time in wheat adapted to north-western European agricultural environments, thereby providing knowledge to aid future development of wheat cultivars adapted to agricultural systems under climate change.

## Supplementary Information

Below is the link to the electronic supplementary material.

Supplementary file1 (DOCX 370 KB)

Supplementary file2 (DOCX 15 KB)

Supplementary file3 (DOCX 150 KB)

Supplementary file4 (XLSX 4202 KB)

## Data Availability

The pedigree of all MAGIC lines along with genotypic information and genetic map are available via https://www.niab.com/research/agricultural-crop-research/resources/niab-magic-population-resources.
