# Supplementary material for: An eight-founder wheat MAGIC population allows fine-mapping of flowering time loci and provides novel insights into the genetic control of flowering time
Source: Theor Appl Genet. 2024 Nov 22;137(12):277. doi: 10.1007/s00122-024-04787-7 (PMC11584503; doi:10.1007/s00122-024-04787-7)
Supplement: Supplementary file 1 — Supplementary file1 (DOCX 370 KB) [file 122_2024_4787_MOESM1_ESM.docx]

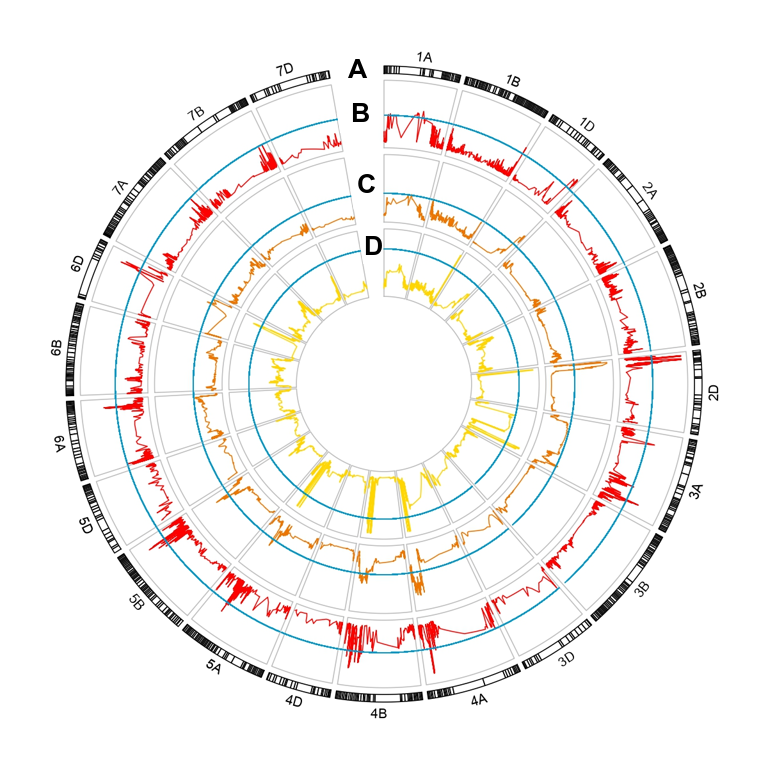


**Supplementary Figure S1.** A comparison of quantitative trait locus (QTL) detection from the three of the genetic analysis methods used, based on the meta-analysis scores for growth stage GS55 (flag leaf blade all visible). (A) The 21 chromosomes of wheat, indicating physical map position of SNPs (based on the wheat reference genome assembly, RefSeq v1.0). QTL scans using the following analysis methods: (B) SNP, (C) identity by descent (IBD), and (D) composite interval mapping with 10 covariates (CIM_cov10). In tracks B-D, the significance threshold is shown in blue.
