# Supplementary material for: An eight-founder wheat MAGIC population allows fine-mapping of flowering time loci and provides novel insights into the genetic control of flowering time
Source: Theor Appl Genet. 2024 Nov 22;137(12):277. doi: 10.1007/s00122-024-04787-7 (PMC11584503; doi:10.1007/s00122-024-04787-7)
Supplement: Supplementary file 2 — Supplementary file2 (DOCX 15 KB) [file 122_2024_4787_MOESM2_ESM.docx]

**Supplementary Text S1**

*Additional candidate genes for QFt.niab-1B.05 and QFt.niab-1D.03*

Additional candidate genes for our MAGIC QTL *QFt.niab-1B.05* (*Eps-B1*) and *QFt.niab-1D.03* (*Eps-D1*) were identified in the reference wheat genome assembly of Chinese Spring. These include: (1) *CO*-like genes: like other Clade 4 CMF genes (Cockram et al. 2012), the *CO*-like genes identified at the homoeologous 1B (*TaCMF6-A1*, *-A2* and *–A3*) and 1D (*TaCMF-D1*, *-D2*) loci contain four exons, with intron III located within the CCT protein domain. CCT domain genes play a well-documented role in the control of flowering time in many monocot and dicot species (reviewed by Li and Xu 2017). However, *OsCCT01* (*OsCMF1*) form rice is the only characterized Clade 4 member to date, with overexpression delaying flowering under short day (SD) and long day (LD) photoperiods (Zhang et al. 2015). (2) Also in the QTL region is a Zinc finger CCCH domain gene. This gene family is known to play a role in many developmental and environmental response pathways, and are known to affect flowering in Arabidopsis (Seok et al. 2016). The rice *Early heading date 4* (*Edh4*) locus, which controls LD and SD photoperiod response independent of the pathway mediated by the CO homologue *Heading date 1* (*Hd1*), encodes a CCCH-type zinc finger protein (Gao et al. 2013).

*Additional candidate genes for QFt.niab-4A.03 and QFt.niab-4B.01*

Three additional candidate genes are considered here: (1) Two tandemly duplicated *FLOWERING PROMOTOR FACTOR 1* (*FPF1*) genes. Transgenic studies have shown that *FPF1* promotes flowering in Arabidopsis (Kania et al. 1997; Melzer et al. 2002) and that heterologous constitutive expression of *FPF1* in rice reduces time to flowering (Xu et al. 2005). (2) MADS box transcription factor *TraesCS4B02G302600* was found to belong to the *SHORT VEGETATIVE PHASE* (*SVP*) family. In Arabidopsis, the SVP family genes *AGAMOUS LIKE 24* (*AGL24*) and SVP regulate floral transition and floral meristem identity via modulation of the MADS-box genes *APETALA 1* (*AP1*), *CAULIFLOWER* (*CAL*) and *FRUITFUL* (*FUL*) (Ferrandiz et al. 2000; Wigge et al. 2005; Grandi et al. 2012; Jaeger et al. 2013). The wheat gene is orthologous to *OsMADS47*, which together with *OsMADS22* and *OsMADS55*, belong to the SPV family in rice. Overexpression of *OsMADS22* induces spikelet meristem indeterminacy (Sentoku et al. 2005), while heterologous constitutive expression of *OsMADS47* and *OsMADS55* in Arabidopsis promotes vegetative development (Fornara et al. 2008).

**Supplementary references**

Ferrandiz C, Gu Q, Martienssen R, Yanofsky MF (2000) Redundant regulation of meristem identity and plant architecture by *FRUITFULL*, *APETALA1* and *CAULIFLOWER*. Development 127: 725-734.

Fornara F, Gregis V, Pelucchi N, Colombo L, Kater M (2008) The rice *StMADS11-*like genes *OsMADS22* and *OsMADS47* cause floral reversions in *Arabidopsis* without complementing the *svp* and *agl24* mutants. Journal of Experimental Botany 59: 2191-2190.

Gao H, Zheng X-M, Fei G, Chen J, Jin M, Ren Y, Wu W, Zhou K, Sheng P, Zhou F, Jiang L, Wang J, Zhang X, Guo X, Wang J-L, Cheng Z, Wu C, Wang H, Wan J-M (2013) *Edh4* encodes a novel and *Oryza*-genus specific regulator of photoperiodic flowering in rice. PLoS Genetic*s* 9: e1003281.

Grandi V, Gregis V, Kater MM (2012) Uncovering genetic and molecular interactions among floral meristem identity genes in *Arabidopsis thaliana*. Plant Journal 69: 881-893.

Jaeger KE, Pullen N, Lamzin S, Morris RJ, Wigge PA (2013) Interlocking feedback loops govern the dynamic behavior of the floral transition in *Arabidopsis*. Plant Cell 25: 820-833.

Kania T, Russenberger D, Peng S, Melzer S (1997) *FPP1* promotes flowering in Arabidopsis. Plant Cell 9: 1327-1338.

Li Y, Xu M (2017) CCT family genes in cereal crops: a current overview. The Crop Journal 6: 449-458.

Melzer S, Kampmann G, Chandler J, Apel K (2002) *FPP1* modulates the competence to flowering in *Arabidopsis*. The Plant Journal 18: 395-405.

Seok H-Y, Woo D-H, Park H-Y, Lee S-Y, Tran HT, Lee E-H, Nguyen LV, Moon Y-H (2016) AtC3H17, a non-tandem CCCH zinc finger protein, functions as a nuclear transcriptional activator and has pleiotropic effects on vegetative development, flowering and seed development in Arabidopsis. Plant Cell and Physiology 57: 603-615.

Wigge PA, Kim MC, Jaeger KE, et al. (2005) Integration of spatial and temporal information during floral induction in *Arabidopsis*. Science 309: 1056-1059.

Sentoku N, Kato H, Kitanyo H, Imai R (2005) *OsMADS22*, an *STMADS11*-like MADS-box gene of rice, is expressed in non-vegetative tissues and its ectopic expression induces spikelet meristem indeterminacy. Molecular Genetics and Genomics 273: 1-9.

Xu M-L, Jiang J-F, Ge L, Xu Y-Y, Chen H, Zhao Y, Bi Y-R, Wen J-Q, Chong K (2005) *FPF1* transgene leads to altered flowering time and root development in rice. Plant Cell Reports 24: 79-85.

Zhang L, Li Q, Dong H, He Q, Liang L, Tan C, Han Z, Yao W, Li G, Zhao H, Xie W, Xing Y (2015) Three CCT domain-containing genes were identified to regulate heading date by candidate gene-based association mapping and transformation in rice. Scientific Reports 5: 7663.
