# Supplementary material for: An eight-founder wheat MAGIC population allows fine-mapping of flowering time loci and provides novel insights into the genetic control of flowering time
Source: Theor Appl Genet. 2024 Nov 22;137(12):277. doi: 10.1007/s00122-024-04787-7 (PMC11584503; doi:10.1007/s00122-024-04787-7)
Supplement: Supplementary file 3 — Supplementary file3 (DOCX 150 KB) [file 122_2024_4787_MOESM3_ESM.docx]

**Supplementary Table S1.** UK MetOffice weather data for the Cambridge trial sites for the month of May, in years 2013-2016. SD = standard deviation. RH = relative humidity. NA = not available.

| **Trial** | **Statistic** | **Air Temp Max (°C)** | **Air Temp Min (°C)** | **Air Temp Max daytime (°C)** | **Air Temp Min daytime (°C)** | **Air Temp Max nightime (°C)** | **Air Temp Min nightime (°C)** | **Soil Temp Min Grass (°C)** | **Air Temp @09:00 (°C)** | **RH% @ 09:00** | **Soil Temp @10cm depth (°C)** | **Rainfall total (mm day^-1^)** | **Rainfall daytime (mm day^-1^)** | **Rainfall nightime (mm day^-1^)** |
| --- | --- | --- | --- | --- | --- | --- | --- | --- | --- | --- | --- | --- | --- | --- |
| 2013 | Mean | 15.9 | 6.1 | 15.8 | 9.9 | 12.2 | 6.2 | 4.3 | 11.5 | 74.2 | NA | 1.7 | 1.0 | 0.7 |
| 2013 | SD | 3.2 | 3.1 | 3.2 | 2.6 | 2.4 | 3.1 | 4.0 | 2.7 | 17.4 | NA | 2.5 | 1.8 | 1.3 |
| 2014 | Mean | 17.7 | 8.6 | 17.7 | 11.9 | 14.1 | 8.7 | 7.3 | 13.8 | 77.0 | 14.0 | 2.7 | 1.1 | 1.7 |
| 2014 | SD | 3.3 | 2.8 | 3.3 | 2.8 | 2.7 | 2.8 | 3.2 | 2.8 | 12.9 | 1.9 | 4.9 | 2.3 | 3.9 |
| 2015 | Mean | 16.5 | 7.5 | 16.5 | 10.6 | 13.2 | 7.5 | 5.1 | 12.5 | 70.3 | 12.7 | 1.0 | 0.6 | 0.5 |
| 2015 | SD | 2.4 | 3.0 | 2.4 | 2.0 | 2.1 | 3.1 | 4.1 | 1.9 | 10.5 | 1.5 | 2.0 | 1.1 | 1.6 |
| 2016 | Mean | 17.7 | 7.8 | 17.7 | 11.7 | 13.9 | 7.8 | 5.7 | 13.6 | 74.0 | 13.8 | 0.9 | 0.7 | 0.2 |
| 2016 | SD | 3.5 | 3.7 | 3.5 | 2.8 | 2.8 | 3.7 | 5.3 | 2.9 | 13.5 | 1.9 | 2.3 | 1.8 | 0.7 |

**Supplementary Table S2.** Final model selections for the trial analysis of p-rep flowering time scores based on experimental design for 2013 and 2014 and meta-analysis of Zadoks growth stage 39 (GS39), GS55 and GS61 across years 2013-2016. The final model was first selected on GS55 and then validated on the other traits for the same year.

| **Trial year** | **Final model** |
| --- | --- |
| 2013 | Trait~row.field+.col.field+Main.Blocks+Main.Blocks:Sub.Blocks+  row.field:col.field+row.field:Main.Blocks+col.field:Main.Blocks+  row.field:col.field:Main.Blocks+row.field:Main.Blocks:Sub.Blocks+  col.field:Main.Blocks:Sub.Blocks+(1\|Line) |
| 2014 | Trait~row.field+col.field+Main.Blocks+ Main.Blocks:Sub.Blocks+  row.field:col.field+row.field:Main.Blocks+col.field:Main.Blocks+  row.field:col.field:Main.Blocks+row.field:Main.Blocks:Sub.Blocks+  col.field:Main.Blocks:Sub.Blocks+row.field:col.field:Main.Blocks:  Sub.Blocks+(1\|Line) |

**Supplementary Table S3.** List of calculated -log_10_(*p*) significance thresholds for all trait-environment combinations, the meta-analysis and validation datasets across all used genetic analysis methods. Genetic analysis methods used: SNP = single marker analysis. SNP_IBD = identity by descent. IM = interval mapping. CIM = composite interval mapping. Additionally, the effects of allelic variation at *Ppd-D1* were included in some models, indicated here as ‘_PPD’. NA = not applicable.

| **Trait** | **SNP** | **SNP_PPD** | **IBD** | **IBD_PPD** | **IM cov0** | **CIM cov2** | **CIM cov10** |
| --- | --- | --- | --- | --- | --- | --- | --- |
| BLUE_2011 | 3.08 | 3.48 | 2.52 | 2.41 | 4.27 | 4.27 | 4.27 |
| BLUE_2012 | 2.82 | 2.65 | 2.40 | 2.14 | 4.17 | 4.17 | 4.17 |
| GS_M2012 | 3.12 | 3.18 | 3.02 | 2.60 | 4.37 | 4.37 | 4.37 |
| GS_J2012 | 3.04 | 3.81 | 3.30 | 3.06 | 4.62 | 4.62 | 4.62 |
| GS39_2013 | 3.00 | 3.53 | 3.70 | 4.90 | 4.09 | 4.09 | 4.09 |
| GS55_2013 | 3.22 | 3.08 | 2.46 | 2.31 | 4.12 | 4.12 | 4.12 |
| GS61_2013 | 3.02 | 3.14 | 2.44 | 2.36 | 4.25 | 4.25 | 4.25 |
| GS39_2014 | 3.17 | 3.09 | 2.87 | 2.30 | 4.26 | 4.26 | 4.26 |
| GS55_2014 | 2.80 | 2.96 | 2.64 | 2.01 | 4.44 | 4.44 | 4.44 |
| GS61_2014 | 3.29 | 3.36 | 2.84 | 2.64 | 4.39 | 4.39 | 4.39 |
| GS39_2015 | 3.70 | 3.76 | 3.52 | 3.61 | 4.31 | 4.31 | 4.31 |
| GS55_2015 | 3.06 | 3.22 | 2.54 | 2.39 | 4.27 | 4.27 | 4.27 |
| GS61_2015 | 2.94 | 3.17 | 2.41 | 2.72 | 4.60 | 4.60 | 4.60 |
| GS39_2016 | 3.37 | 3.14 | 3.12 | 2.71 | 4.49 | 4.49 | 4.49 |
| GS55_2016 | 8.45 | 3.79 | 3.56 | 2.60 | 4.24 | 4.24 | 4.24 |
| GS61_2016 | 3.22 | 3.99 | 3.27 | 3.32 | 4.35 | 4.35 | 4.35 |
| GS39_meta | 2.94 | 2.90 | 2.83 | 2.24 | 4.52 | 4.52 | 4.52 |
| GS55_meta | 2.87 | 2.98 | 2.47 | 2.07 | 4.47 | 4.47 | 4.47 |
| GS61_meta | 2.96 | 3.02 | 2.86 | 2.37 | 4.61 | 4.61 | 4.61 |
| HOH_2015 | 3.32 | 3.79 | 2.88 | 2.70 | NA | NA | NA |

**Supplementary Table S4.** List of the 57 quantitative trait loci (QTL) detected across trait-environment combinations and the growth stage meta-analyses including the QTL interval chromosome (Chr), start marker name (Start SNP), start position on the genetic map of Gardner *et al*., (2016) (Start cM), start position bin (Start bin) and end marker name (End SNP), end position on the genetic map (End cM), end position bin (End bin). NA = not applicable. Regions of very low genetic recombination as identified in the MAGIC population by Gardner et al. (2016) with which QTL span or overlap: ^*^ pericentromeric region, ^†^ wheat/rye 1B/1R chromosomal introgression and the pericentromeric region.

| **Chr** | **QTL ID** | **Start SNP** | **Start cM** | **Start bin (Mbp)** | **End SNP** | **End cM** | **End bin (Mbp)** |
| --- | --- | --- | --- | --- | --- | --- | --- |
| 1A | *QFt.niab-1A.01*^*^ | BS00029346_51 | 55.6 | 37.5 | BobWhite_c46349_402 | 75.1 | 354.9 |
| 1A | *QFt.niab-1A.02* | Excalibur_c15357_432 | 111.8 | 499.4 | wsnp_BG314157A_Ta_2_1 | 113.8 | 503.4 |
| 1A | *QFt.niab-1A.03* | wsnp_BG263358A_Ta_2_1 | 147.1 | 531.6 | wsnp_BG263358A_Ta_2_1 | 147.1 | 531.6 |
| 1A | *QFt.niab-1A.04* | BS00106641_51 | 208.9 | 577.1 | wsnp_Ku_c14496_22838796 | 207.9 | 578.6 |
| 1B | *QFt.niab-1B.01* | BS00071161_51 | 1 | 1.5 | Excalibur_c3270_1566 | 33.4 | 22.8 |
| 1B | *QFt.niab-1B.02*^†^ | RAC875_c10659_1539 | 88.5 | 94.8 | BS00067244_51 | 82.5 | 401.8 |
| 1B | *QFt.niab-1B.03* | Tdurum_contig60052_184 | 168.3 | 473.5 | Tdurum_contig8158_269 | 196.7 | 564.2 |
| 1B | *QFt.niab-1B.04* | BS00075247_51 | 207 | 566.8 | BobWhite_c16543_993 | 239.3 | 626.1 |
| 1B | *QFt.niab-1B.05* | BobWhite_c11756_79 | 335.3 | 676.8 | BS00001128_51 | 350.1 | 689.5 |
| 1D | *QFt.niab-1D.01* | BobWhite_c33756_74 | 19.3 | 10.4 | RAC875_c57095_240 | 19.8 | 11.7 |
| 1D | *QFt.niab-1D.02* | BS00060042_51 | 69.2 | 339.3 | BS00060042_51 | 69.2 | 339.3 |
| 1D | *QFt.niab-1D.03* | BS00063028_51 | 122.2 | 460.6 | Excalibur_c17004_1098 | 127.8 | 495.2 |
| 2A | *QFt.niab-2A.01* | IAAV2585 | 59.4 | 36.6 | IAAV2585 | 59.4 | 36.6 |
| 2A | *QFt.niab-2A.02* | IAAV7468 | 78.9 | 46.1 | IAAV7468 | 78.9 | 46.1 |
| 2A | *QFt.niab-2A.03* | wsnp_Ex_c35331_43499339 | 86.5 | 48.9 | wsnp_Ex_c35331_43499339 | 86.5 | 48.9 |
| 2A | *QFt.niab-2A.04* | Kukri_c9118_1891 | 159 | 709.9 | Tdurum_contig33398_106 | 160.5 | 718.9 |
| 2B | *QFt.niab-2B.01*^*^ | Kukri_c34353_821 | 180.9 | 165.5 | BS00029146_51 | 198.8 | 249.4 |
| 2B | *QFt.niab-2B.02* | wsnp_Ex_rep_c103064_88104690 | 290.6 | 751.3 | CAP11_c1820_244 | 333.5 | 782.5 |
| 2B | *QFt.niab-2B.03* | BS00056642_51 | 370.4 | 793.1 | BS00056645_51 | 370.9 | 793.1 |
| 2D | *QFt.niab-2D.01* | D_GDEEGVY02IF3IH_412 | 27.5 | 9.6 | wsnp_Ex_c12947_20510337 | 96.3 | 83.2 |
| 2D | *QFt.niab-2D.02* | Excalibur_rep_c115179_438 | 164.9 | 570.9 | Excalibur_c61922_195 | 160.9 | NA |
| 3A | *QFt.niab-3A.01*^*^ | Kukri_c4568_1708 | 67.9 | 34.5 | RAC875_rep_c90531_283 | 216.4 | 689.5 |
| 4A | *QFt.niab-4A.01*^*^ | BobWhite_c5633_59 | 63.7 | 38.4 | Tdurum_contig34920_104 | 80.0 | 125.4 |
| 4A | *QFt.niab-4A.02* | RAC875_c27704_420 | 87.6 | 570.5 | RAC875_c1022_3059 | 98.2 | 596.0 |
| 4A | *QFt.niab-4A.03* | IAAV7132 | 124.6 | 613.6 | Excalibur_c10699_404 | 214.7 | 744.3 |
| 4B | *QFt.niab-4B.01*^*^ | BS00105308_51 | 73.6 | 139.8 | wsnp_BE403378B_Ta_2_1 | 206.0 | 659.2 |
| 4D | *QFt.niab-4D.01* | Excalibur_c26088_184 | 9.9 | 1.2 | wsnp_Ex_c683_1341113 | 48.5 | 54.4 |
| 4D | *QFt.niab-4D.02* | D_GDEEGVY01C7BQU_446 | 83.7 | 455.7 | BS00047797_51 | 84.2 | 456.1 |
| 5A | *QFt.niab-5A.01* | BS00030991_51 | 35.1 | 19.3 | BS00030991_51 | 35.1 | 19.3 |
| 5A | *QFt.niab-5A.02*^*^ | wsnp_BE444644A_Ta_2_1 | 63.6 | 158.2 | BS00046529_51 | 84.6 | 434.7 |
| 5A | *QFt.niab-5A.03* | BS00022495_51 | 128.2 | 480.2 | BobWhite_c48730_723 | 199.8 | 573.7 |
| 5A | *QFt.niab-5A.04* | Ex_c27046_1546 | 214 | 574.7 | Ku_c12469_837 | 225.1 | 596.5 |
| 5A | *QFt.niab-5A.05* | BS00068108_51 | 310 | 702.5 | Ex_c2171_2600 | 310.0 | 708.4 |
| 5B | *QFt.niab-5B.01* | Kukri_c52_225 | 113.4 | 539.3 | Kukri_c52_225 | 113.4 | 539.3 |
| 5B | *QFt.niab-5B.02* | BS00068710_51 | 154.2 | 558.1 | GENE_2794_534 | 191.1 | 580.8 |
| 5B | *QFt.niab-5B.03* | BS00022673_51 | 199.3 | 584.1 | BobWhite_c12883_272 | 230.7 | 647.9 |
| 5B | *QFt.niab-5B.04* | BS00048316_51 | 265 | 679.7 | RAC875_c3420_537 | 268.1 | 679.8 |
| 5B | *QFt.niab-5B.05* | RAC875_c22722_671 | 289.6 | 693.6 | BS00077733_51 | 289.1 | 693.9 |
| 5D | *QFt.niab-5D.01* | Ku_c13618_473 | 0 | 29.6 | RAC875_rep_c74985_362 | 0.5 | 29.2 |
| 5D | *QFt.niab-5D.02* | BS00082423_51 | 120.8 | 489.8 | wsnp_Ex_c23618_32855041 | 153.4 | 540.3 |
| 5D | *QFt.niab-5D.03* | Kukri_c45387_380 | 186.6 | 549.6 | Kukri_c17093_1099 | 171.1 | 562.7 |
| 6A | *QFt.niab-6A.01* | RAC875_c68978_220 | 13.2 | 5.3 | Excalibur_c20597_509 | 13.2 | 6.7 |
| 6A | *QFt.niab-6A.02* | BS00023192_51 | 48.9 | 13.8 | Kukri_c14679_913 | 46.9 | 15.3 |
| 6A | *QFt.niab-6A.03*^*^ | Kukri_c214QFt.niab-5A.057 | 122.4 | 71.4 | RFL_Contig5037_560 | 210.6 | 594.7 |
| 6B | *QFt.niab-6B.01* | BobWhite_c36228_53 | 145.4 | 485.9 | BS00035381_51 | 141.9 | 542.2 |
| 6B | *QFt.niab-6B.02* | Ra_c69293_781 | 183.1 | 664 | IACX2322 | 206.4 | 694.2 |
| 6D | *QFt.niab-6D.01*^*^ | Excalibur_c10358_1800 | 25.8 | 1.8 | BS00070856_51 | 215.0 | 472.8 |
| 7A | *QFt.niab-7A.01* | BobWhite_c10229_415 | 217.8 | 244.5 | Excalibur_c200_1664 | 217.8 | 244.5 |
| 7A | *QFt.niab-7A.02* | Ku_c29856_174 | 274.2 | 616.4 | TA006231_0789 | 279.2 | 650.6 |
| 7A | *QFt.niab-7A.03* | IACX2471 | 305.7 | 669.7 | IACX2471 | 305.7 | 669.7 |
| 7A | *QFt.niab-7A.04* | BobWhite_c149_3064 | 306.7 | 670.8 | IAAV6957 | 314.8 | 675.2 |
| 7A | *QFt.niab-7A.05* | Excalibur_c1142_724 | 346.6 | 700.3 | Excalibur_c1142_724 | 346.6 | 700.3 |
| 7B | *QFt.niab-7B.01* | BS00022127_51 | 9.4 | 3.5 | Excalibur_c26611_325 | 44.3 | 58.8 |
| 7B | *QFt.niab-7B.02* | GENE_1477_748 | 83.7 | 104.3 | wsnp_JD_c646_966400 | 84.2 | 109.0 |
| 7B | *QFt.niab-7B.03* | Kukri_c15912_860 | 168.1 | 674 | Kukri_c15912_860 | 168.1 | 674.0 |
| 7B | *QFt.niab-7B.04* | IACX11443 | 184.8 | NA | IACX11443 | 184.8 | NA |
| 7B | *QFt.niab-7B.05* | Tdurum_contig8402_460 | 226.2 | 711.2 | CAP12_c580_105 | 273.6 | 750.6 |

**Supplementary Table S5.** Identified peak markers for the 57 identified quantitative trait loci (QTL), including marker name (Peak SNP), genetic map position based on the map published by Gardner et al*.* (2016) (Peak cM) and genetic map bin (Peak bin). NA = not applicable.

| **Chr** | **QTL ID** | **Peak SNP name** | **Peak cM** | **Peak bin (Mbp)** |
| --- | --- | --- | --- | --- |
| 1A | *QFt.niab-1A.01* | RAC875_c11899_366 | 63.5 | 58.7 |
| 1A | *QFt.niab-1A.02* | wsnp_BG314157A_Ta_2_1 | 113.8 | 503.4 |
| 1A | *QFt.niab-1A.03* | wsnp_BG263358A_Ta_2_1 | 147.1 | 531.6 |
| 1A | *QFt.niab-1A.04* | BS00106641_51 | 208.9 | 577.1 |
| 1B | *QFt.niab-1B.01* | Excalibur_c21898_1423 | 1.5 | 1.4 |
| 1B | *QFt.niab-1B.02* | BS00031948_51 | 90.6 | 119.9 |
| 1B | *QFt.niab-1B.03* | BS00048610_51 | 186.1 | 543.1 |
| 1B | *QFt.niab-1B.04* | BS00066338_51 | 231.7 | 588.8 |
| 1B | *QFt.niab-1B.05* | IAAV5516 | 349.6 | 688.7 |
| 1D | *QFt.niab-1D.01* | RAC875_c57095_240 | 19.8 | 11.7 |
| 1D | *QFt.niab-1D.02* | BS00060042_51 | 69.2 | 339.3 |
| 1D | *QFt.niab-1D.03* | Kukri_c29687_369 | 123.2 | 493.2 |
| 2A | *QFt.niab-2A.01* | IAAV2585 | 59.4 | 36.6 |
| 2A | *QFt.niab-2A.02* | IAAV7468 | 78.9 | 46.1 |
| 2A | *QFt.niab-2A.03* | wsnp_Ex_c35331_43499339 | 86.5 | 48.9 |
| 2A | *QFt.niab-2A.04* | BS00037160_51 | 159.0 | 718.6 |
| 2B | *QFt.niab-2B.01* | wsnp_RFL_Contig2506_2098552 | 177.9 | 183.7 |
| 2B | *QFt.niab-2B.02* | BS00000012_51 | 320.3 | 776.8 |
| 2B | *QFt.niab-2B.03* | BS00056645_51 | 370.9 | 793.1 |
| 2D | *QFt.niab-2D.01* | Ppd_D1 | 60.7 | 34.0 |
| 2D | *QFt.niab-2D.02* | RAC875_c11373_804 | 162.4 | 590.7 |
| 3A | *QFt.niab-3A.01* | IAAV4781 | 98.0 | 107.7 |
| 4A | *QFt.niab-4A.01* | wsnp_Ku_c14515_22860258 | 79.0 | 103.4 |
| 4A | *QFt.niab-4A.02* | BS00010339_51 | 91.1 | 584.0 |
| 4A | *QFt.niab-4A.03* | BS00064140_51 | 153.2 | 666.1 |
| 4B | *QFt.niab-4B.01* | Excalibur_c22632_576 | 165.6 | 609.4 |
| 4D | *QFt.niab-4D.01* | RAC875_c1673_663 | 32.2 | 16.6 |
| 4D | *QFt.niab-4D.02* | BS00023112_51 | 83.7 | 455.8 |
| 5A | *QFt.niab-5A.01* | BS00030991_51 | 35.1 | 19.3 |
| 5A | *QFt.niab-5A.02* | wsnp_Ex_c26581_35828388 | 62.6 | 317.2 |
| 5A | *QFt.niab-5A.03* | BS00068178_51 | 169.8 | 540.1 |
| 5A | *QFt.niab-5A.04* | tplb0038h19_1394 | 225.6 | 585.4 |
| 5A | *QFt.niab-5A.05* | BS00023138_51 | 310.0 | 704.5 |
| 5B | *QFt.niab-5B.01* | Kukri_c52_225 | 113.4 | 539.3 |
| 5B | *QFt.niab-5B.02* | TA002756_0960 | 154.7 | 558.3 |
| 5B | *QFt.niab-5B.03* | BobWhite_c22572_782 | 215.5 | 618.1 |
| 5B | *QFt.niab-5B.04* | BS00010168_51 | 265.5 | 680.3 |
| 5B | *QFt.niab-5B.05* | BS00077733_51 | 289.1 | 693.9 |
| 5D | *QFt.niab-5D.01* | Ku_c13618_473 | 0 | 29.6 |
| 5D | *QFt.niab-5D.02* | BS00033770_51 | 124.3 | 496.8 |
| 5D | *QFt.niab-5D.03* | D_contig27392_286 | 183.6 | 552.8 |
| 6A | *QFt.niab-6A.01* | RAC875_c68978_220 | 13.2 | 5.3 |
| 6A | *QFt.niab-6A.02* | Kukri_c14679_913 | 46.9 | 15.3 |
| 6A | *QFt.niab-6A.03* | Ku_c69999_111 | 172.4 | 552.5 |
| 6B | *QFt.niab-6B.01* | Excalibur_c20083_433 | 142.4 | 537.5 |
| 6B | *QFt.niab-6B.02* | BS00037933_51 | 190.2 | 668.8 |
| 6D | *QFt.niab-6D.01* | IAAV8527 | 117.7 | 389.6 |
| 7A | *QFt.niab-7A.01* | BobWhite_c10229_415 | 217.8 | 244.5 |
| 7A | *QFt.niab-7A.02* | RAC875_c27696_2299 | 277.2 | 644.6 |
| 7A | *QFt.niab-7A.03* | IACX2471 | 305.7 | 669.7 |
| 7A | *QFt.niab-7A.04* | IAAV6957 | 314.8 | 675.2 |
| 7A | *QFt.niab-7A.05* | Excalibur_c1142_724 | 346.6 | 700.3 |
| 7B | *QFt.niab-7B.01* | Excalibur_c29698_76 | 27.0 | 15.0 |
| 7B | *QFt.niab-7B.02* | GENE_1477_748 | 83.7 | 104.3 |
| 7B | *QFt.niab-7B.03* | Kukri_c15912_860 | 168.1 | 674 |
| 7B | *QFt.niab-7B.04* | IACX11443 | 184.8 | NA |
| 7B | *QFt.niab-7B.05* | RAC875_c525_1885 | 273.1 | 750.1 |

**Supplementary Table S6.** Examples of predicted founder effects on days to flowering at each of the ten ‘major’ flowering time quantitative trait loci (QTL). Results shown are from composite interval mapping using two covariates (CIM_cov2). Genetic map position is according to that if the NIAB Elite MAGIC genetic map (Gardner et al*.* 2016). SE = standard error. NA = not applicable.

| **QTL** | **Trait** | **Chr** | **Pos (cM)** | **Left Mrk** | **Right Mrk** | **Effect Alchemy** | **SE** | **Effect Brompton** | **SE** | **Effect Claire** | **SE** | **Effect Hereward** | **SE** | **Effect Rialto** | **SE** | **Effect Robigus** | **SE** | **Effect Soissons** | **SE** | **Effect Xi19** | **SE** |
| --- | --- | --- | --- | --- | --- | --- | --- | --- | --- | --- | --- | --- | --- | --- | --- | --- | --- | --- | --- | --- | --- |
| *QFt.niab.1B.05* | GS55_2013.2 | 1B | 349.6 | RAC875_c102886_73 | IAAV5516 | -1.0 | 0.5 | 0.1 | 0.4 | -0.2 | 0.5 | 0.3 | 0.4 | 0.1 | 0.5 | -0.0 | 0.5 | -1.4 | 0.4 | 0 | NA |
| *QFt.niab.1D.03* | GS39_2016.3 | 1D | 126.8 | RAC875_c41914_613 | Kukri_c44781_236 | 1.6 | 1.8 | 2.7 | 0.9 | -2.9 | 1.8 | 2.9 | 1.9 | 0.5 | 0.8 | 5.6 | 1.8 | 2.1 | 0.5 | 0 | NA |
| *QFt.niab.2D.01* | GS55_2013.6 | 2D | 60.7 | Kukri_c27309_590 | Ppd_D1 | -0.0 | 0.5 | 0.6 | 0.4 | -0.7 | 0.5 | 0.5 | 0.5 | -0.1 | 0.4 | 0.5 | 0.5 | -3.1 | 0.4 | 0 | NA |
| *QFt.niab.3A.01* | BLUE_2011.7 | 3A | 99.5 | Kukri_c10977_990 | Excalibur_c2419_531 | -0.0 | 0.2 | -0.7 | 0.2 | -0.4 | 0.2 | 0.1 | 0.2 | -0.3 | 0.2 | 0.0 | 0.2 | 0.1 | 0.2 | 0 | NA |
| *QFt.niab.4A.03* | GS55_2015.10 | 4A | 154.2 | BS00064140_51 | RAC875_rep_c109924_289 | 3.1 | 0.6 | 2.5 | 0.7 | 0.8 | 0.6 | 2.4 | 0.6 | 2.0 | 0.5 | 2.1 | 0.5 | 2.3 | 0.7 | 0 | NA |
| *QFt.niab.4B.01* | GS61_2015.11 | 4B | 165.6 | BS00003421_51 | BS00057153_51 | -1.7 | 0.5 | 0.0 | 0.4 | -0.3 | 0.5 | 0.2 | 0.4 | 0.1 | 0.4 | -0.1 | 0.4 | -0.4 | 0.4 | 0 | NA |
| *QFt.niab.4D.01* | GS39_2013.12 | 4D | 32.2 | RAC875_c1673_663 | RHT2 | -1.7 | 1.9 | 0.2 | 0.6 | 1.8 | 1.9 | -0.0 | 0.5 | 0.3 | 0.5 | -1.1 | 0.4 | -1.2 | 0.4 | 0 | NA |
| *QFt.niab.6A.03* | GS39_2013_blup.16 | 6A | 210.6 | TA001855_0472 | BS00062771_51 | -1.1 | 0.4 | -0.3 | 0.3 | 0.9 | 0.4 | 0.2 | 0.3 | -0.8 | 0.3 | -0.1 | 0.3 | 0.5 | 0.3 | 0 | NA |
| *QFt.niab.6D.01* | BLUE_2012.18 | 6D | 142.5 | CAP11_rep_c6864_291 | GENE_4060_219 | 0.6 | 0.3 | -0.4 | 0.1 | -0.2 | 0.2 | -1.1 | 0.3 | NA | NA | 0.1 | 0.2 | -0.1 | 0.2 | 0 | NA |
| *QFt.niab.7B.01* | GS61_2013.20 | 7B | 18.7 | BS00022127_51 | Kukri_c67849_109 | 0.3 | 0.3 | -3.3 | 1.3 | 0.1 | 0.3 | 0.2 | 0.3 | 3.1 | 1.2 | 0.2 | 0.4 | -1.6 | 0.3 | 0 | NA |

**Supplementary Table S7.** Proportion of trait variation explained by the 10 ‘major’ quantitative trait loci (QTL) detected in all analyses. Listed are the trait-environment and meta-analyses they were detected in, the significance value for each analysis and the proportion of variation explained (%var). Genetic analysis methods used: SNP = single marker analysis. SNP_IBD = identity by descent. IM = interval mapping. CIM = composite interval mapping. Additionally, the effects of allelic variation at *Ppd-D1* were included in some models, indicated here as ‘_PPD’. NA = not applicable.

|  |  | **SNP** | | **SNP_PPD** | | **IBD** | | **IBD_PPD** | | **SIM cov0** | | **CIM cov2** | | **CIM cov10** | |
| --- | --- | --- | --- | --- | --- | --- | --- | --- | --- | --- | --- | --- | --- | --- | --- |
| **QTL** | **Trait** | **Sign** | **%var** | **Sign** | **%var** | **Sign** | **%var** | **Sign** | **%var** | **Sign** | **%var** | **Sign*** | **%var** | **Sign*** | **%var** |
| *QFt.niab-1B.05* | BLUE_2011 |  |  | 0.90 | 32.99 |  |  | 0.89 | 35.43 |  |  |  |  |  |  |
|  | BLUE_2012 | 0.49 | 1.23 | 2.15 | 35.78 |  |  | 2.57 | 37.75 |  |  | 1.32 | 1.44 | 1.22 | 1.44 |
|  | GS_M2012 |  |  | 0.79 | 32.01 |  |  | 2.52 | 34.94 |  |  |  |  |  |  |
|  | GS_J2012 | 1.17 | 2.31 | 1.20 | 14.10 | 1.47 | 4.14 | 3.98 | 18.65 | 0.15 | 4.11 | 3.28 | 4.11 | 3.60 | 4.11 |
|  | GS39_2013 | 0.59 | 1.82 | 0.78 | 11.53 |  |  |  |  |  |  |  |  |  |  |
|  | GS55_2013 | 0.23 | 2.04 | 1.27 | 18.25 |  |  | 1.11 | 20.92 |  |  | 1.43 | 1.96 | 1.68 | 1.96 |
|  | GS61_2013 | 0.33 | 1.42 | 0.31 | 18.12 |  |  |  |  |  |  |  |  |  |  |
|  | GS39_2014 |  |  | 0.58 | 48.66 |  |  | 0.56 | 49.47 |  |  |  |  | 1.63 | 0.14 |
|  | GS55_2014 |  |  | 1.28 | 42.23 |  |  | 2.61 | 44.35 |  |  | 0.97 | 1.41 | 3.61 | 1.41 |
|  | GS61_2014 |  |  | 2.77 | 53.57 |  |  | 3.46 | 55.81 |  |  | 1.98 | 0.70 | 4.48 | 0.70 |
|  | GS39_2015 |  |  |  |  |  |  |  |  |  |  |  |  | 1.17 | 0.15 |
|  | GS55_2015 |  |  | 0.39 | 28.97 |  |  | 0.65 | 31.13 |  |  |  |  |  |  |
|  | GS61_2015 | 0.54 | 1.34 | 1.66 | 26.76 |  |  | 1.53 | 29.41 |  |  | 0.26 | 1.51 | 0.95 | 1.51 |
|  | GS39_2016 |  |  | 1.15 | 22.18 |  |  | 0.55 | 24.7 |  |  |  |  | 1.87 | 1.11 |
|  | GS55_2016 |  |  | 0.37 | 15.84 |  |  | 0.74 | 19.71 |  |  |  |  | 1.80 | 2.15 |
|  | GS61_2016 | 0.64 | 1.76 | 0.52 | 19.66 |  |  |  |  |  |  |  |  | 0.21 | 1.36 |
|  | GS39_meta | 0.38 | 1.14 | 2.57 | 40.65 |  |  | 2.29 | 42.36 |  |  | 0.98 | 0.74 | 2.53 | 0.74 |
|  | GS55_meta | 0.12 | 1.50 | 2.32 | 35.23 |  |  | 3.27 | 37.77 |  |  | 2.26 | 2.90 | 4.30 | 2.90 |
|  | GS61_meta | 0.19 | 1.65 | 4.74 | 43.35 |  |  | 4.19 | 45.83 |  |  | 1.96 | 1.49 | 4.57 | 1.49 |
| *QFt.niab-1D.03* | BLUE_2011 | 0.17 | 1.66 |  |  | 0.67 | 2.87 |  |  |  |  |  |  |  |  |
|  | BLUE_2012 | 1.92 | 2.73 | 0.56 | 36.95 | 2.28 | 4.45 | 1.15 | 40.19 | 0.76 | 3.63 |  |  | 0.56 | 3.27 |
|  | GS_M2012 | 0.24 | 1.80 |  |  |  |  |  |  |  |  |  |  |  |  |
|  | GS_J2012 | 0.68 | 2.20 |  |  |  |  |  |  |  |  |  |  |  |  |
|  | GS39_2013 | 0.42 | 1.95 |  |  |  |  |  |  |  |  |  |  |  |  |
|  | GS55_2013 |  |  |  |  |  |  |  |  |  |  |  |  |  |  |
|  | GS61_2013 |  |  |  |  |  |  |  |  |  |  |  |  |  |  |
|  | GS39_2014 | 1.63 | 2.66 | 0.57 | 50.01 | 0.73 | 3.76 | 0.32 | 51.78 |  |  |  |  |  |  |
|  | GS55_2014 | 1.47 | 2.41 |  |  | 1.25 | 4.08 |  |  |  |  |  |  |  |  |
|  | GS61_2014 | 0.17 | 1.79 |  |  |  |  |  |  |  |  |  |  |  |  |
|  | GS39_2015 |  |  |  |  |  |  |  |  |  |  |  |  |  |  |
|  | GS55_2015 | 0.26 | 1.86 |  |  | 0.74 | 3.87 |  |  |  |  |  |  |  |  |
|  | GS61_2015 | 0.57 | 2.03 |  |  | 0.80 | 3.83 |  |  |  |  |  |  |  |  |
|  | GS39_2016 | 2.96 | 3.86 | 2.44 | 24.35 | 2.85 | 5.23 | 1.59 | 28.39 | 1.62 | 4.67 |  |  | 1.58 | 4.65 |
|  | GS55_2016 |  |  |  |  |  |  |  |  |  |  |  |  |  |  |
|  | GS61_2016 | 0.39 | 2.08 |  |  |  |  |  |  |  |  |  |  |  |  |
|  | GS39_meta | 2.45 | 3.13 | 1.36 | 42.38 | 1.58 | 4.39 | 1.39 | 45.2 | 0.19 | 3.99 | 0.14 | 3.79 |  |  |
|  | GS55_meta | 1.27 | 2.35 |  |  | 1.45 | 4.09 |  |  |  |  |  |  |  |  |
|  | GS61_meta | 0.92 | 2.13 |  |  | 0.14 | 3.37 |  |  |  |  |  |  |  |  |
| *QFt.niab-2D.01* | BLUE_2011 | 12.93 | 32.77 |  |  | 13.48 | 9.26 |  |  | 45.50 | 27.57 | 51.12 | 27.57 | 47.42 | 27.57 |
|  | BLUE_2012 | 13.18 | 36.74 |  |  | 13.61 | 12.97 |  |  | 52.79 | 3.20 | 58.99 | 3.20 | 8.96 | 3.20 |
|  | GS_M2012 | 12.88 | 32.4 |  |  | 12.98 | 10.85 |  |  | 46.35 | 28.28 | 48.75 | 28.28 | 66.72 | 28.28 |
|  | GS_J2012 | 12.97 | 13.97 |  |  | 8.92 | 6.03 |  |  | 1.86 | 11.34 | 14.39 | 11.34 | 2.62 | 11.34 |
|  | GS39_2013 | 12.26 | 11.67 |  |  | 8.47 | 4.89 |  |  | 8.79 | 9.65 | 11.84 | 9.65 | 16.36 | 9.65 |
|  | GS55_2013 | 12.78 | 18.29 |  |  | 13.54 | 6.34 |  |  | 16.87 | 14.43 | 18.51 | 14.43 | 26.86 | 14.43 |
|  | GS61_2013 | 12.98 | 18.66 |  |  | 13.56 | 7.16 |  |  | 18.28 | 15.30 | 22.14 | 15.30 | 34.12 | 15.30 |
|  | GS39_2014 | 12.83 | 49.71 |  |  | 13.13 | 19.43 |  |  | 93.23 | 42.26 | 96.34 | 42.26 | 112.67 | 42.26 |
|  | GS55_2014 | 13.20 | 42.80 |  |  | 13.36 | 16.46 |  |  | 72.52 | 36.78 | 8.38 | 36.78 | 13.83 | 36.78 |
|  | GS61_2014 | 12.78 | 55.49 |  |  | 13.16 | 19.45 |  |  | 11.71 | 46.84 | 122.0 | 46.84 | 145.77 | 46.84 |
|  | GS39_2015 | 12.33 | 32.87 |  |  | 12.48 | 12.16 |  |  | 49.37 | 29.14 | 51.75 | 29.14 | 69.14 | 29.14 |
|  | GS55_2015 | 12.94 | 30.6 |  |  | 13.46 | 12.44 |  |  | 37.56 | 24.47 | 43.42 | 24.47 | 53.83 | 24.47 |
|  | GS61_2015 | 13.64 | 28.63 |  |  | 13.59 | 11.83 |  |  | 32.23 | 22.29 | 36.43 | 22.29 | 55.23 | 22.29 |
|  | GS39_2016 | 12.63 | 22.17 |  |  | 12.88 | 8.74 |  |  | 25.19 | 19.20 | 26.47 | 19.20 | 32.38 | 19.20 |
|  | GS55_2016 | 7.55 | 16.76 |  |  | 12.44 | 6.58 |  |  | 13.96 | 12.87 | 15.62 | 12.87 | 19.87 | 12.87 |
|  | GS61_2016 | 12.78 | 20.9 |  |  | 12.73 | 7.58 |  |  | 18.94 | 15.70 | 21.54 | 15.70 | 33.32 | 15.70 |
|  | GS39_meta | 13.57 | 41.18 |  |  | 13.17 | 15.45 |  |  | 69.51 | 35.77 | 77.36 | 35.77 | 15.52 | 35.77 |
|  | GS55_meta | 13.13 | 35.91 |  |  | 13.53 | 13.46 |  |  | 51.84 | 29.96 | 59.24 | 29.96 | 81.26 | 29.96 |
|  | GS61_meta | 13.42 | 44.89 |  |  | 13.14 | 15.88 |  |  | 74.58 | 37.28 | 84.95 | 37.28 | 121.77 | 37.28 |
| *QFt.niab-3A.01* | BLUE_2011 | 0.30 | 1.46 | 0.49 | 33.21 | 0.71 | 3.64 | 1.55 | 34.14 |  |  | 0.77 | 3.34 | 2.26 | 2.91 |
|  | BLUE_2012 | 2.18 | 2.79 | 3.28 | 37.04 | 0.98 | 3.64 | 2.60 | 37.89 | 0.67 | 4.90 | 1.90 | 4.70 | 7.63 | 4.90 |
|  | GS_M2012 | 2.65 | 3.60 | 4.48 | 32.40 | 1.29 | 4.84 | 3.80 | 35.52 | 0.13 | 4.50 | 1.63 | 4.50 |  |  |
|  | GS_J2012 | 0.23 | 1.88 |  |  |  |  |  |  |  |  |  |  |  |  |
|  | GS39_2013 |  |  |  |  |  |  |  |  |  |  |  |  | 3.78 | 2.83 |
|  | GS55_2013 | 0.95 | 2.57 | 1.38 | 18.33 | 2.11 | 4.70 | 3.46 | 20.28 | 0.85 | 4.83 | 3.17 | 4.83 | 6.32 | 4.83 |
|  | GS61_2013 | 1.34 | 1.45 | 0.76 | 18.69 | 2.96 | 4.40 | 2.73 | 19.76 | 0.42 | 3.99 | 0.65 | 3.65 | 2.35 | 3.99 |
|  | GS39_2014 | 0.99 | 2.03 | 1.47 | 49.80 | 0.12 | 2.69 | 0.49 | 50.97 |  |  | 0.26 | 2.19 | 0.35 | 1.99 |
|  | GS55_2014 | 0.95 | 1.84 | 1.37 | 43.09 | 0.26 | 2.84 | 1.52 | 43.74 |  |  | 0.31 | 2.44 | 1.43 | 2.38 |
|  | GS61_2014 | 0.12 | 1.24 | 2.18 | 54.38 | 0.22 | 2.30 | 0.37 | 54.76 |  |  | 0.58 | 2.26 | 0.54 | 2.25 |
|  | GS39_2015 |  |  | 0.63 | 32.22 |  |  |  |  |  |  |  |  |  |  |
|  | GS55_2015 | 0.11 | 1.35 | 2.72 | 29.13 |  |  | 1.14 | 30.31 |  |  |  |  | 0.83 | 2.11 |
|  | GS61_2015 | 0.15 | 0.98 | 0.63 | 26.92 |  |  | 0.37 | 27.85 |  |  |  |  |  |  |
|  | GS39_2016 | 0.83 | 1.56 | 1.79 | 22.02 | 1.20 | 1.44 | 2.46 | 22.54 | 0.85 | 3.97 | 1.97 | 3.84 | 0.85 | 3.84 |
|  | GS55_2016 |  |  | 0.61 | 15.79 |  |  | 1.14 | 16.92 |  |  |  |  | 0.67 | 2.11 |
|  | GS61_2016 |  |  |  |  |  |  |  |  |  |  |  |  |  |  |
|  | GS39_meta | 0.48 | 1.79 | 0.63 | 41.36 |  |  | 2.23 | 42.18 |  |  | 0.97 | 2.13 | 1.51 | 2.94 |
|  | GS55_meta | 0.91 | 2.02 | 1.12 | 35.62 | 0.15 | 2.73 | 0.95 | 36.29 |  |  | 0.24 | 2.72 | 2.25 | 2.72 |
|  | GS61_meta | 0.63 | 1.38 | 2.68 | 43.94 | 0.59 | 2.42 | 0.64 | 44.29 |  |  |  |  | 1.13 | 1.87 |
| *QFt.niab-4A.03* | BLUE_2011 | 1.54 | 2.52 | 3.70 | 32.37 | 0.44 | 2.21 | 2.95 | 33.74 |  |  | 1.65 | 2.40 | 0.66 | 2.40 |
|  | BLUE_2012 | 2.62 | 3.29 | 6.83 | 36.54 | 1.95 | 4.40 | 6.53 | 37.64 | 0.16 | 3.75 | 4.28 | 3.72 | 6.22 | 3.70 |
|  | GS_M2012 | 0.39 | 1.93 | 2.63 | 31.57 |  |  | 1.62 | 32.64 |  |  |  |  |  |  |
|  | GS_J2012 | 4.11 | 4.34 | 5.73 | 14.19 | 0.65 | 4.01 | 3.23 | 15.67 |  |  | 0.95 | 3.44 | 0.97 | 3.26 |
|  | GS39_2013 | 0.35 | 2.00 | 0.44 | 12.03 |  |  |  |  |  |  |  |  |  |  |
|  | GS55_2013 | 2.92 | 3.94 | 4.37 | 18.78 | 2.26 | 4.99 | 4.45 | 20.55 | 0.88 | 4.25 | 3.33 | 3.93 | 5.29 | 4.22 |
|  | GS61_2013 | 3.62 | 4.36 | 4.82 | 18.64 | 2.86 | 5.55 | 5.95 | 19.93 | 0.79 | 4.58 | 3.75 | 4.26 | 4.81 | 4.15 |
|  | GS39_2014 |  |  | 0.96 | 48.83 |  |  | 0.93 | 49.89 |  |  |  |  |  |  |
|  | GS55_2014 | 1.18 | 2.11 | 5.22 | 42.67 | 1.42 | 4.26 | 6.78 | 44.02 | 0.40 | 4.12 | 4.14 | 3.46 | 3.55 | 3.46 |
|  | GS61_2014 |  |  | 3.47 | 53.80 |  |  | 4.19 | 54.29 |  |  | 0.64 | 1.51 | 0.96 | 1.51 |
|  | GS39_2015 |  |  | 1.35 | 31.79 |  |  | 0.97 | 33.08 |  |  |  |  | 0.51 | 2.13 |
|  | GS55_2015 | 2.63 | 3.40 | 4.60 | 29.16 | 1.92 | 4.35 | 3.99 | 30.36 | 0.53 | 4.60 | 4.12 | 4.10 | 5.37 | 3.17 |
|  | GS61_2015 | 1.49 | 2.60 | 2.76 | 27.02 | 0.41 | 3.15 | 2.23 | 28.10 |  |  | 1.00 | 2.98 | 1.95 | 2.98 |
|  | GS39_2016 |  |  | 1.77 | 22.21 | 0.42 | 2.19 | 1.59 | 25.23 |  |  |  |  |  |  |
|  | GS55_2016 |  |  | 1.51 | 16.52 |  |  | 1.28 | 18.31 |  |  | 0.27 | 2.97 | 1.12 | 2.96 |
|  | GS61_2016 | 0.42 | 2.14 | 1.61 | 20.24 |  |  | 0.84 | 22.52 |  |  | 0.50 | 3.00 |  |  |
|  | GS39_meta | 0.87 | 1.63 | 3.22 | 40.69 |  |  | 2.62 | 41.92 |  |  |  |  |  |  |
|  | GS55_meta | 2.66 | 3.19 | 6.39 | 35.59 | 2.46 | 4.69 | 6.86 | 37.17 | 0.67 | 4.11 | 4.99 | 3.91 | 6.40 | 3.91 |
|  | GS61_meta | 1.95 | 2.89 | 6.81 | 43.69 | 0.58 | 3.75 | 7.24 | 44.89 |  |  | 4.42 | 2.81 | 7.76 | 3.44 |
| *QFt.niab-4B.01* | BLUE_2011 | 0.39 | 2.30 | 0.34 | 32.79 | 0.05 | 2.94 |  |  |  |  |  |  |  |  |
|  | BLUE_2012 | 2.49 | 3.56 | 5.30 | 36.55 | 1.69 | 4.58 | 2.38 | 36.61 |  |  | 0.96 | 3.10 | 1.17 | 3.10 |
|  | GS_M2012 |  |  | 0.33 | 31.96 |  |  | 0.12 | 32.94 |  |  |  |  |  |  |
|  | GS_J2012 | 0.16 | 1.73 |  |  |  |  |  |  |  |  |  |  |  |  |
|  | GS39_2013 | 0.83 | 2.67 | 0.80 | 11.78 |  |  |  |  |  |  | 0.35 | NA | 0.46 | 2.21 |
|  | GS55_2013 | 2.15 | 3.97 | 3.55 | 18.53 | 0.73 | 3.99 | 1.38 | 19.04 | 0.87 | NA | 0.77 | 3.36 | 3.16 | 3.00 |
|  | GS61_2013 | 4.55 | 5.46 | 6.15 | 19.16 | 2.96 | 5.77 | 3.69 | 20.11 | 1.14 | 4.87 | 2.32 | 4.41 | 5.75 | 4.64 |
|  | GS39_2014 | 0.99 | 1.86 | 0.85 | 48.91 |  |  | 0.00 | 49.85 |  |  |  |  |  |  |
|  | GS55_2014 | 0.55 | 2.32 | 2.19 | 42.24 | 0.84 | 2.85 | 0.64 | 42.08 |  |  |  |  | 0.84 | 2.62 |
|  | GS61_2014 |  |  | 1.97 | 53.66 |  |  | 0.20 | 53.46 |  |  |  |  | 0.79 | 1.60 |
|  | GS39_2015 |  |  |  |  |  |  |  |  |  |  |  |  |  |  |
|  | GS55_2015 | 1.70 | 3.12 | 2.72 | 29.37 | 0.49 | 3.82 | 0.52 | 30.08 |  |  |  |  | 0.57 | 2.85 |
|  | GS61_2015 | 3.18 | 4.18 | 3.71 | 27.12 | 1.24 | 4.47 | 0.73 | 28.45 |  |  |  |  | 1.31 | 3.70 |
|  | GS39_2016 |  |  | 0.21 | 21.96 |  |  |  |  |  |  |  |  | 0.11 | 1.27 |
|  | GS55_2016 |  |  |  |  |  |  |  |  |  |  |  |  |  |  |
|  | GS61_2016 |  |  |  |  |  |  |  |  |  |  |  |  |  |  |
|  | GS39_meta | 0.68 | 2.27 | 2.15 | 40.92 | 0.14 | 3.14 | 0.18 | 41.79 |  |  |  |  |  |  |
|  | GS55_meta | 1.86 | 3.22 | 3.62 | 35.20 | 0.33 | 3.57 | 1.46 | 35.22 |  |  |  |  | 2.55 | 2.72 |
|  | GS61_meta | 2.53 | 3.40 | 4.39 | 43.57 |  |  | 0.96 | 43.68 |  |  |  |  | 0.82 | 2.10 |
| *QFt.niab-4D.01* | BLUE_2011 |  |  |  |  |  |  |  |  |  |  |  |  |  |  |
|  | BLUE_2012 |  |  | 0.57 | 35.77 |  |  |  |  |  |  |  |  |  |  |
|  | GS_M2012 |  |  |  |  |  |  |  |  |  |  |  |  |  |  |
|  | GS_J2012 |  |  |  |  |  |  |  |  |  |  |  |  |  |  |
|  | GS39_2013 | 5.12 | 5.32 | 6.94 | 11.50 | 0.76 | 4.77 | 1.37 | 15.98 | 0.63 | NA | 1.76 | 3.31 | 6.86 | 3.31 |
|  | GS55_2013 | 0.91 | 1.74 | 1.78 | 18.11 |  |  |  |  |  |  |  |  |  |  |
|  | GS61_2013 |  |  |  |  |  |  |  |  |  |  |  |  |  |  |
|  | GS39_2014 |  |  | 2.36 | 48.72 | 0.26 | 2.69 | 1.87 | 51.19 |  |  |  |  |  |  |
|  | GS55_2014 |  |  |  |  |  |  |  |  |  |  |  |  |  |  |
|  | GS61_2014 |  |  |  |  |  |  |  |  |  |  |  |  |  |  |
|  | GS39_2015 |  |  |  |  |  |  |  |  |  |  |  |  |  |  |
|  | GS55_2015 |  |  |  |  |  |  |  |  |  |  |  |  |  |  |
|  | GS61_2015 |  |  |  |  |  |  |  |  |  |  |  |  |  |  |
|  | GS39_2016 | 0.48 | 1.76 | 1.77 | 21.86 |  |  | 1.77 | 24.75 |  |  |  |  | 0.22 | 1.98 |
|  | GS55_2016 |  |  |  |  |  |  |  |  |  |  |  |  |  |  |
|  | GS61_2016 |  |  |  |  |  |  |  |  |  |  |  |  |  |  |
|  | GS39_meta | 0.66 | 2.24 | 5.26 | 40.54 |  |  | 2.51 | 43.40 |  |  |  |  | 2.22 | 1.90 |
|  | GS55_meta |  |  |  |  |  |  |  |  |  |  |  |  |  |  |
|  | GS61_meta |  |  |  |  |  |  |  |  |  |  |  |  |  |  |
| *QFt.niab-6A.03* | BLUE_2011 | 0.63 | 1.40 | 1.36 | 32.50 |  |  | 0.42 | 35.34 |  |  |  |  |  |  |
|  | BLUE_2012 | 0.26 | 1.77 | 1.48 | 36.04 |  |  | 0.24 | 37.63 |  |  |  |  |  |  |
|  | GS_M2012 | 1.75 | 3.15 | 3.18 | 32.82 |  |  | 1.52 | 36.54 |  |  |  |  |  |  |
|  | GS_J2012 | 0.59 | 2.15 | 0.62 | 14.77 |  |  |  |  |  |  |  |  |  |  |
|  | GS39_2013 | 2.47 | 3.50 | 1.23 | 13.00 | 0.57 | 3.82 |  |  | 1.31 | 4.34 | 1.33 | 4.34 | 0.32 | NA |
|  | GS55_2013 | 1.46 | 2.94 | 1.37 | 18.29 | 0.29 | 3.62 | 1.40 | 20.43 |  |  |  |  |  |  |
|  | GS61_2013 | 0.64 | 1.76 |  |  |  |  |  |  |  |  |  |  |  |  |
|  | GS39_2014 |  |  |  |  |  |  |  |  |  |  |  |  |  |  |
|  | GS55_2014 | 0.39 | 2.06 | 0.64 | 42.34 |  |  |  |  |  |  |  |  |  |  |
|  | GS61_2014 |  |  |  |  |  |  |  |  |  |  |  |  |  |  |
|  | GS39_2015 |  |  | 0.11 | 32.44 |  |  |  |  |  |  |  |  |  |  |
|  | GS55_2015 | 0.89 | 2.46 | 1.20 | 29.24 | 0.62 | 3.37 | 0.43 | 30.97 |  |  |  |  |  |  |
|  | GS61_2015 | 0.49 | 2.06 | 0.48 | 27.14 | 0.56 | 2.58 | 0.26 | 28.84 |  |  |  |  |  |  |
|  | GS39_2016 | 1.90 | 2.74 | 0.76 | 22.77 |  |  |  |  |  |  |  |  |  |  |
|  | GS55_2016 |  |  |  |  |  |  |  |  |  |  |  |  |  |  |
|  | GS61_2016 |  |  |  |  |  |  |  |  |  |  |  |  |  |  |
|  | GS39_meta | 1.46 | 2.67 | 2.16 | 41.17 |  |  | 0.57 | 41.63 |  |  |  |  |  |  |
|  | GS55_meta | 1.34 | 2.59 | 1.79 | 35.31 |  |  | 0.52 | 36.75 |  |  |  |  |  |  |
|  | GS61_meta | 0.13 | 1.71 | 0.52 | 43.48 |  |  |  |  |  |  |  |  |  |  |
| *QFt.niab-6D.01* | BLUE_2011 | 2.28 | 3.27 | 0.53 | 32.34 | 1.87 | 4.00 | 0.70 | 34.74 | 1.34 | 4.51 | 0.19 | 4.37 | 1.52 | 4.38 |
|  | BLUE_2012 | 2.54 | 3.12 | 2.63 | 35.79 | 0.55 | 3.38 | 0.77 | 37.14 | 0.56 | 3.85 | 0.16 | 3.43 | 1.69 | 3.85 |
|  | GS_M2012 | 2.58 | 3.34 | 0.99 | 31.61 |  |  | 0.13 | 34.20 | 0.56 | NA |  |  |  |  |
|  | GS_J2012 | 0.57 | 2.44 |  |  |  |  |  |  |  |  |  |  |  |  |
|  | GS39_2013 |  |  |  |  |  |  |  |  |  |  |  |  |  |  |
|  | GS55_2013 | 0.39 | 1.84 | 1.29 | 18.61 |  |  | 0.43 | 20.12 |  |  |  |  |  |  |
|  | GS61_2013 |  |  |  |  |  |  |  |  |  |  |  |  |  |  |
|  | GS39_2014 |  |  | 0.87 | 48.97 |  |  |  |  |  |  |  |  |  |  |
|  | GS55_2014 | 0.66 | 2.01 | 1.57 | 42.31 |  |  | 0.18 | 42.91 |  |  |  |  |  |  |
|  | GS61_2014 |  |  | 0.22 | 54.63 |  |  |  |  |  |  |  |  |  |  |
|  | GS39_2015 |  |  |  |  |  |  |  |  |  |  |  |  |  |  |
|  | GS55_2015 | 3.16 | 4.16 | 2.39 | 28.88 | 1.37 | 4.36 | 0.59 | 30.86 | 0.82 | 4.14 |  |  |  |  |
|  | GS61_2015 | 2.71 | 3.68 | 1.48 | 26.75 | 0.99 | 3.87 |  |  | 0.16 | 3.9 |  |  |  |  |
|  | GS39_2016 |  |  | 0.18 | 21.91 |  |  |  |  |  |  |  |  |  |  |
|  | GS55_2016 |  |  | 0.53 | 15.63 | 0.95 | 3.42 | 2.58 | 17.70 | 0.13 | 3.74 |  |  |  |  |
|  | GS61_2016 | 0.36 | 2.09 |  |  |  |  |  |  |  |  |  |  |  |  |
|  | GS39_meta | 0.20 | 1.80 | 0.99 | 40.57 |  |  |  |  |  |  |  |  |  |  |
|  | GS55_meta | 2.25 | 3.15 | 2.25 | 34.98 | 0.43 | 3.51 | 0.78 | 36.4 | 0.26 | 3.85 |  |  | 0.94 | 3.85 |
|  | GS61_meta | 1.56 | 2.36 | 1.12 | 43.64 |  |  |  |  |  |  |  |  |  |  |
| *QFt.niab-7B.01* | BLUE_2011 |  |  |  |  | 0.38 | 3.21 |  |  |  |  |  |  | 0.16 | 2.35 |
|  | BLUE_2012 |  |  | 0.89 | 35.94 | 0.16 | 3.18 | 0.66 | 38.56 |  |  | 1.20 | 2.66 | 0.16 | 2.97 |
|  | GS_M2012 |  |  |  |  |  |  |  |  |  |  |  |  |  |  |
|  | GS_J2012 |  |  |  |  | 0.39 | 4.20 |  |  |  |  |  |  | 1.17 | 3.23 |
|  | GS39_2013 |  |  |  |  |  |  |  |  |  |  |  |  |  |  |
|  | GS55_2013 |  |  |  |  |  |  |  |  |  |  |  |  |  |  |
|  | GS61_2013 | 2.28 | 3.24 | 3.94 | 18.50 | 0.56 | 4.37 | 1.23 | 21.98 | 0.39 | 4.31 | 2.15 | 4.25 | 2.93 | 4.31 |
|  | GS39_2014 |  |  | 0.23 | 48.73 |  |  |  |  |  |  |  |  |  |  |
|  | GS55_2014 |  |  |  |  |  |  |  |  |  |  |  |  |  |  |
|  | GS61_2014 |  |  | 0.48 | 53.91 |  |  |  |  |  |  |  |  |  |  |
|  | GS39_2015 |  |  | 1.15 | 32.27 |  |  |  |  |  |  |  |  |  |  |
|  | GS55_2015 |  |  | 0.17 | 28.92 |  |  |  |  |  |  |  |  |  |  |
|  | GS61_2015 | 0.67 | 2.26 | 1.42 | 26.94 |  |  | 0.37 | 28.77 |  |  |  |  |  |  |
|  | GS39_2016 |  |  |  |  |  |  |  |  |  |  |  |  |  |  |
|  | GS55_2016 |  |  |  |  |  |  |  |  |  |  |  |  |  |  |
|  | GS61_2016 |  |  |  |  |  |  |  |  |  |  |  |  |  |  |
|  | GS39_meta |  |  | 0.41 | 40.88 |  |  |  |  |  |  |  |  |  |  |
|  | GS55_meta |  |  |  |  |  |  |  |  |  |  |  |  |  |  |
|  | GS61_meta |  |  | 2.37 | 43.68 |  |  | 0.88 | 45.36 |  |  | 0.27 | 2.61 | 0.52 | 2.00 |

**Supplementary Table S8.** Proportion of trait variation explained by the remaining 47 ‘minor’ quantitative trait loci (QTL) which were not detected in all analyses, including the trait-environment and meta-analyses they were detected in, the significance value for each analysis and the proportion of variation explained. Only trait-environments or meta-analyses in which significant effects were detected are presented. Genetic analysis methods used: SNP = single marker analysis. SNP_IBD = identity by descent. IM = interval mapping. CIM = composite interval mapping. Additionally, the effects of allelic variation at *Ppd-D1* were included in some models, indicated here as ‘_PPD’. NA = not applicable.

|  |  | **SNP** | | **SNP_PPD** | | **IBD** | | **IBD_PPD** | | **SIM cov0** | | **CIM cov2** | | **CIM cov10** | |
| --- | --- | --- | --- | --- | --- | --- | --- | --- | --- | --- | --- | --- | --- | --- | --- |
| **QTL** | **Trait** | **Sign** | **%var** | **Sign** | **%var** | **Sign** | **%var** | **Sign** | **%var** | **Sign** | **%var** | **Sign** | **%var** | **Sign** | **%var** |
| *QFt.niab-1A.01* | GS61_2013 | 0.35 | 2.09 |  |  | 0.62 | 2.78 |  |  |  |  |  |  |  |  |
|  | GS55_2014 | 0.42 | 2.18 | 0.63 | 42.56 |  |  |  |  |  |  |  |  |  |  |
|  | GS61_2014 | 0.48 | 1.93 | 1.19 | 53.84 |  |  |  |  |  |  |  |  |  |  |
|  | GS55_meta | 0.17 | 1.94 |  |  | 0.82 | 2.50 |  |  |  |  |  |  |  |  |
|  | GS61_meta | 0.12 | 1.73 | 0.12 | 43.55 |  |  |  |  |  |  |  |  |  |  |
| *QFt.niab-1A.02* | GS_J2012 | 0.15 | 1.17 |  |  |  |  |  |  |  |  |  |  |  |  |
|  | GS39_2014 | 0.12 | 2.09 |  |  |  |  |  |  |  |  |  |  |  |  |
|  | GS39_meta | 0.39 | 1.91 |  |  |  |  |  |  |  |  |  |  |  |  |
| *QFt.niab-1A.03* | GS61_2013 | 0.74 | 1.82 |  |  |  |  |  |  |  |  |  |  |  |  |
| *QFt.niab-1A.04* | GS39_2013 |  |  |  |  |  |  |  |  |  |  |  |  | 0.67 | 0.29 |
| *QFt.niab-1B.01* | GS39_2013 |  |  |  |  |  |  |  |  |  |  |  |  | 1.92 | 2.70 |
|  | GS61_2015 | 0.22 | 0.04 |  |  | 0.15 | 3.46 | 0.26 | 29.67 |  |  |  |  |  |  |
|  | GS55_meta |  |  |  |  | 0.45 | 3.48 |  |  |  |  |  |  |  |  |
| *QFt.niab-1B.02* | BLUE_2012 |  |  |  |  |  |  | 0.32 | 37.89 |  |  |  |  |  |  |
|  | GS39_2014 |  |  |  |  |  |  | 0.25 | 49.84 |  |  |  |  |  |  |
|  | GS55_2015 |  |  |  |  |  |  | 0.55 | 31.83 |  |  |  |  |  |  |
|  | GS61_2015 |  |  |  |  |  |  | 0.32 | 29.51 |  |  |  |  |  |  |
|  | GS39_meta |  |  | 0.13 | 40.99 |  |  |  |  |  |  |  |  |  |  |
|  | GS55_meta |  |  |  |  |  |  | 0.16 | 36.69 |  |  |  |  |  |  |
| *QFt.niab-1B.03* | BLUE_2012 |  |  | 0.30 | 36.29 |  |  | 0.92 | 38.21 |  |  |  |  |  |  |
|  | GS39_2014 |  |  | 0.37 | 48.84 |  |  | 0.86 | 50.13 |  |  |  |  |  |  |
|  | GS55_2014 |  |  | 0.60 | 42.59 |  |  | 0.86 | 44.19 |  |  |  |  |  |  |
|  | GS55_2015 |  |  |  |  |  |  | 0.60 | 31.36 |  |  |  |  |  |  |
|  | GS61_2014 |  |  | 0.20 | 54.18 |  |  | 0.56 | 55.47 |  |  |  |  |  |  |
|  | GS39_meta |  |  | 0.46 | 40.67 |  |  | 0.48 | 42.75 |  |  |  |  |  |  |
|  | GS55_meta |  |  | 0.58 | 35.22 |  |  | 0.90 | 37.03 |  |  |  |  |  |  |
|  | GS61_meta |  |  | 0.18 | 43.88 |  |  | 0.75 | 45.10 |  |  |  |  |  |  |
| *QFt.niab-1B.04* | BLUE_2012 |  |  | 0.52 | 36.31 |  |  |  |  |  |  |  |  |  |  |
|  | GS39_2014 |  |  | 0.86 | 49.13 |  |  |  |  |  |  |  |  |  |  |
|  | GS55_2014 |  |  |  |  |  |  | 0.16 | 43.48 |  |  |  |  |  |  |
|  | GS61_2014 |  |  | 0.28 | 53.97 |  |  |  |  |  |  |  |  |  |  |
|  | GS55_2015 |  |  | 0.63 | 29.49 |  |  | 0.47 | 30.4 |  |  |  |  |  |  |
|  | GS39_2016 |  |  | 0.55 | 24.07 |  |  |  |  |  |  |  |  |  |  |
|  | GS55_meta |  |  | 0.33 | 35.47 |  |  | 0.39 | 36.37 |  |  |  |  |  |  |
|  | GS61_meta |  |  |  |  |  |  | 0.26 | 44.49 |  |  |  |  |  |  |
| *QFt.niab-1D.01* | BLUE_2011 |  |  |  |  |  |  | 0.46 | 35.47 |  |  |  |  | 0.20 | 2.1 |
| *QFt.niab-1D.02* | GS55_2013 | 0.15 | 2.00 |  |  |  |  |  |  |  |  |  |  |  |  |
| *QFt.niab-2A.01* | GS39_2016 | 0.12 | 2.09 |  |  |  |  |  |  |  |  |  |  |  |  |
|  | GS61_2015 | 0.19 | 1.78 |  |  |  |  |  |  |  |  |  |  |  |  |
| *QFt.niab-2A.02* | GS_J2012 |  |  |  |  |  |  | 0.62 | 18.26 |  |  |  |  |  |  |
| *QFt.niab-2A.03* | GS_J2012 | 0.51 | 1.51 |  |  |  |  |  |  |  |  |  |  |  |  |
| *QFt.niab-2A.04* | GS39_2014 |  |  | 0.23 | 48.66 |  |  | 0.11 | 50.20 |  |  |  |  |  |  |
|  | GS39_meta |  |  | 0.93 | 40.93 |  |  |  |  |  |  |  |  |  |  |
| *QFt.niab-2B.01* | GS_J2012 | 0.33 | 2.26 |  |  |  |  | 0.12 | 17.28 |  |  |  |  |  |  |
|  | GS39_2013 | 0.57 | 2.28 |  |  |  |  |  |  |  |  |  |  |  |  |
| *QFt.niab-2B.02* | BLUE_2011 | 2.76 | 3.16 |  |  | 0.16 | 3.55 | 0.55 | 34.55 |  |  |  |  |  |  |
|  | GS55_2015 |  |  |  |  | 0.18 | 2.65 |  |  |  |  |  |  | 0.31 | 2.68 |
| *QFt.niab-2B.03* | GS55_2015 |  |  |  |  |  |  | 0.16 | 31.68 |  |  |  |  |  |  |
| *QFt.niab-2D.02* | BLUE_2012 | 0.23 | 1.68 | 0.33 | 37.04 |  |  |  |  |  |  |  |  |  |  |
|  | GS61_2013 | 0.20 | 1.79 |  |  |  |  |  |  |  |  |  |  |  |  |
| *QFt.niab-4A.01* | BLUE_2012 | 0.26 | 1.09 | 1.63 | 36.6 |  |  | 0.31 | 37.15 |  |  |  |  |  |  |
|  | GS55_2014 |  |  | 0.20 | 42.48 |  |  |  |  |  |  |  |  |  |  |
|  | GS55_2015 |  |  | 0.56 | 29.57 |  |  |  |  |  |  |  |  |  |  |
|  | GS55_2016 |  |  |  |  |  |  | 0.51 | 19.4 |  |  |  |  |  |  |
|  | GS55_meta | 0.48 | 1.24 | 1.37 | 35.81 |  |  | 0.24 | 36.63 |  |  |  |  |  |  |
| *QFt.niab-4A.02* | BLUE_2011 |  |  | 1.46 | 32.61 | 0.56 | 2.86 | 1.70 | 34.49 |  |  |  |  |  |  |
|  | BLUE_2012 |  |  | 0.57 | 35.72 |  |  | 0.67 | 37.17 |  |  |  |  |  |  |
|  | GS_M2012 | 0.45 | 0.74 | 0.23 | 31.64 | 0.23 | 3.52 | 1.65 | 33.80 |  |  | 0.22 | NA | 0.67 | 1.44 |
|  | GS_J2012 | 0.25 | 2.00 | 0.11 | 13.76 |  |  | 0.25 | 16.41 |  |  |  |  |  |  |
|  | GS61_2016 | 0.25 | 0.32 | 0.19 | 19.68 |  |  |  |  |  |  |  |  | 0.45 | 2.30 |
| *QFt.niab-4D.02* | GS61_2013 |  |  |  |  |  |  | 0.14 | 21.72 |  |  |  |  |  |  |
|  | GS61_meta |  |  |  |  |  |  | 0.60 | 45.35 |  |  |  |  |  |  |
| *QFt.niab-5A.01* | GS55_2013 |  |  | 0.80 | 19.64 |  |  |  |  |  |  |  |  |  |  |
| *QFt.niab-5A.02* | BLUE_2012 |  |  | 0.33 | 36.49 |  |  |  |  |  |  |  |  |  |  |
|  | GS61_2013 | 0.48 | 2.11 | 0.78 | 19.94 |  |  |  |  |  |  |  |  |  |  |
|  | GS55_2015 |  |  | 0.12 | 29.53 |  |  |  |  |  |  |  |  |  |  |
| *QFt.niab-5A.03* | BLUE_2012 | 0.82 | 1.27 | 3.53 | 36.00 |  |  | 2.18 | 38.87 |  |  | 0.66 | 2.63 | 0.34 | 2.96 |
|  | GS55_2013 |  |  | 0.78 | 18.63 |  |  | 0.30 | 21.87 |  |  |  |  |  |  |
|  | GS39_2014 |  |  | 0.98 | 49.06 |  |  |  |  |  |  |  |  |  |  |
|  | GS55_2014 | 0.18 | 1.02 | 2.67 | 41.98 |  |  | 0.38 | 43.82 |  |  | 0.16 | 2.62 | 0.72 | 2.31 |
|  | GS61_2014 |  |  | 0.66 | 53.71 |  |  | 0.76 | 55 |  |  |  |  | 1.27 | 2.37 |
|  | GS55_2015 | 1.63 | 1.61 | 2.66 | 29.72 | 0.36 | 3.63 | 2.74 | 33.09 |  |  | 1.39 | 3.40 | 0.74 | 3.40 |
|  | GS61_2015 | 0.51 | 1.27 | 1.19 | 27.21 | 1.54 | 4.01 | 2.96 | 30.61 |  |  | 1.16 | 3.59 | 1.17 | 3.35 |
|  | GS39_2016 |  |  | 1.60 | 22.70 |  |  | 1.26 | 24.47 |  |  |  |  |  |  |
|  | GS55_2016 |  |  | 1.43 | 16.40 |  |  | 1.82 | 19.25 |  |  |  |  | 0.65 | 3.14 |
|  | GS61_2016 |  |  | 0.31 | 20.20 | 0.43 | 4.35 | 1.82 | 23.43 |  |  | 0.56 | 3.33 | 1.39 | 3.33 |
|  | GS39_meta |  |  | 2.88 | 41.41 |  |  | 1.38 | 43.12 |  |  |  |  | 1.32 | 1.6 |
|  | GS55_meta | 0.17 | 1.58 | 4.47 | 35.30 | 0.39 | 3.53 | 2.86 | 38.09 |  |  | 1.41 | 3.50 | 2.49 | 3.50 |
|  | GS61_meta |  |  | 2.75 | 43.67 | 0.36 | 3.42 | 2.49 | 45.99 |  |  | 1.13 | 3.27 | 3.75 | 2.97 |
| *QFt.niab-5A.04* | BLUE_2012 |  |  | 0.85 | 37.12 |  |  |  |  |  |  |  |  |  |  |
|  | GS_M2012 |  |  | 1.55 | 33.63 |  |  | 0.29 | 35.81 |  |  |  |  | 0.60 | 2.47 |
|  | GS39_2014 |  |  | 0.27 | 49.24 |  |  |  |  |  |  |  |  |  |  |
|  | GS55_2014 |  |  | 1.28 | 43.35 |  |  |  |  |  |  |  |  | 0.85 | 1.10 |
|  | GS61_2014 |  |  | 0.13 | 54.85 |  |  |  |  |  |  |  |  |  |  |
|  | GS61_2016 |  |  |  |  |  |  |  |  |  |  |  |  | 0.27 | 0.67 |
|  | GS39_meta |  |  | 0.36 | 41.03 |  |  |  |  |  |  |  |  |  |  |
|  | GS55_meta |  |  |  |  |  |  |  |  |  |  |  |  | 0.86 | 0.75 |
|  | GS61_meta |  |  |  |  |  |  |  |  |  |  |  |  | 0.91 | 0.66 |
| *QFt.niab-5A.05* | GS55_2014 | 0.43 | 2.20 | 0.57 | 42.01 |  |  | 0.31 | 44.66 |  |  |  |  |  |  |
|  | GS55_2015 |  |  | 0.86 | 29.88 |  |  |  |  |  |  |  |  |  |  |
|  | GS55_meta |  |  | 0.14 | 35.19 |  |  |  |  |  |  |  |  |  |  |
| *QFt.niab-5B.01* | GS_M2012 | 0.58 | 1.53 |  |  |  |  |  |  |  |  |  |  |  |  |
| *QFt.niab-5B.02* | BLUE_2011 | 1.36 | 0.32 |  |  |  |  |  |  |  |  |  |  |  |  |
|  | GS55_2014 | 0.46 | 1.49 |  |  |  |  | 0.17 | 43.78 |  |  |  |  |  |  |
|  | GS55_meta | 0.32 | 1.40 |  |  |  |  |  |  |  |  |  |  |  |  |
|  | GS61_2014 | 0.26 | 1.24 |  |  |  |  |  |  |  |  |  |  |  |  |
|  | GS61_meta | 0.25 | 1.47 |  |  |  |  |  |  |  |  |  |  |  |  |
| *QFt.niab-5B.03* | BLUE_2011 | 1.77 | 3.09 |  |  | 2.73 | 4.37 | 0.15 | 34.41 | 1.67 | 4.93 |  |  |  |  |
|  | BLUE_2012 | 1.24 | 2.39 |  |  | 0.94 | 3.53 |  |  | 0.13 | 3.67 |  |  |  |  |
|  | GS39_2013 | 0.36 | 2.00 |  |  |  |  |  |  |  |  |  |  |  |  |
|  | GS39_2015 | 0.96 | 2.79 |  |  |  |  |  |  |  |  |  |  |  |  |
|  | GS39_meta | 1.49 | 2.72 |  |  | 0.53 | 3.30 |  |  |  |  |  |  |  |  |
|  | GS55_2014 | 1.75 | 3.05 |  |  | 1.43 | 4.33 | 0.16 | 43.19 | 0.75 | 4.39 |  |  |  |  |
|  | GS55_2015 | 2.18 | 3.27 |  |  | 0.91 | 3.11 |  |  |  |  |  |  |  |  |
|  | GS55_meta | 1.99 | 3.12 |  |  | 1.53 | 4.00 |  |  | 0.36 | 4.90 |  |  |  |  |
|  | GS61_2014 | 1.53 | 3.26 |  |  | 1.77 | 4.08 |  |  | 0.47 | 4.23 |  |  |  |  |
|  | GS61_2015 | 1.98 | 3.07 |  |  | 0.88 | 3.35 |  |  |  |  |  |  |  |  |
|  | GS61_2016 | 0.40 | 2.22 |  |  |  |  |  |  |  |  |  |  |  |  |
|  | GS61_meta | 2.25 | 3.35 |  |  | 0.79 | 3.62 |  |  | 0.22 | 3.93 |  |  |  |  |
| *QFt.niab-5B.04* | GS55_2015 | 0.22 | 2.03 |  |  |  |  |  |  |  |  |  |  |  |  |
| *QFt.niab-5B.05* | BLUE_2012 | 0.35 | 1.82 |  |  |  |  |  |  |  |  |  |  |  |  |
|  | GS61_meta | 0.32 | 1.83 |  |  |  |  |  |  |  |  |  |  |  |  |
| *QFt.niab-5D.01* | BLUE_2012 |  |  | 0.35 | 36.92 |  |  | 0.29 | 37.83 |  |  |  |  |  |  |
|  | GS55_meta |  |  |  |  |  |  | 0.48 | 37.19 |  |  |  |  |  |  |
| *QFt.niab-5D.02* | BLUE_2011 |  |  |  |  |  |  | 0.56 | 34.75 |  |  |  |  |  |  |
|  | GS55_2014 |  |  |  |  |  |  | 0.68 | 43.45 |  |  |  |  |  |  |
|  | GS61_2014 |  |  |  |  |  |  | 0.19 | 55.01 |  |  |  |  |  |  |
|  | GS55_2015 |  |  |  |  |  |  | 0.92 | 31.19 |  |  |  |  |  |  |
|  | GS55_meta |  |  |  |  |  |  | 0.64 | 36.56 |  |  |  |  |  |  |
|  | GS61_meta |  |  |  |  |  |  | 0.14 | 45.25 |  |  |  |  |  |  |
| *QFt.niab-5D.03* | BLUE_2012 |  |  | 0.52 | 36.29 |  |  | 0.74 | 36.59 |  |  |  |  |  |  |
|  | GS55_2014 |  |  | 0.95 | 42.92 |  |  | 0.65 | 43.72 |  |  |  |  |  |  |
| *QFt.niab-6A.01* | GS39_2014 | 0.74 | 2.51 |  |  |  |  |  |  |  |  |  |  |  |  |
|  | GS55_2014 | 0.82 | 2.23 |  |  |  |  |  |  |  |  |  |  |  |  |
|  | GS61_2014 | 0.34 | 2.22 |  |  |  |  |  |  |  |  |  |  |  |  |
| *QFt.niab-6A.02* | BLUE_2012 |  |  | 0.91 | 36.89 |  |  |  |  |  |  |  |  |  |  |
| *QFt.niab-6B.01* | GS55_2014 |  |  |  |  |  |  | 0.15 | 44.66 |  |  |  |  |  |  |
|  | GS55_meta |  |  |  |  |  |  | 0.28 | 37.79 |  |  |  |  |  |  |
| *QFt.niab-6B.02* | GS39_2013 | 0.57 | 2.14 | 1.12 | 13.86 |  |  |  |  |  |  |  |  |  |  |
|  | GS55_2013 |  |  | 0.83 | 19.34 |  |  |  |  |  |  |  |  |  |  |
|  | GS61_2013 |  |  | 0.86 | 19.87 |  |  |  |  |  |  |  |  |  |  |
|  | GS61_2015 |  |  | 0.12 | 27.84 |  |  |  |  |  |  |  |  |  |  |
| *QFt.niab-7A.01* | BLUE_2012 |  |  |  |  | 0.68 | 2.97 |  |  |  |  |  |  |  |  |
| *QFt.niab-7A.02* | GS55_2013 |  |  |  |  |  |  |  |  |  |  |  |  | 1.76 | 2.13 |
|  | GS61_2016 | 0.32 | 0.17 |  |  |  |  |  |  |  |  |  |  |  |  |
| *QFt.niab-7A.03* | GS55_2013 |  |  |  |  |  |  | 0.7 | 21.4 |  |  |  |  |  |  |
| *QFt.niab-7A.04* | GS55_2014 |  |  | 1.39 | 43.25 |  |  |  |  |  |  |  |  |  |  |
|  | GS55_meta |  |  | 0.79 | 35.95 |  |  |  |  |  |  |  |  |  |  |
| *QFt.niab-7A.05* | GS61_2015 |  |  | 0.53 | 28.41 |  |  |  |  |  |  |  |  |  |  |
| *QFt.niab-7B.02* | GS61_2015 | 0.67 | 1.90 |  |  |  |  |  |  |  |  |  |  |  |  |
| *QFt.niab-7B.03* | BLUE_2012 | 0.49 | 1.80 |  |  |  |  |  |  |  |  |  |  |  |  |
|  | GS39_2013 | 1.88 | 2.91 |  |  |  |  |  |  |  |  |  |  |  |  |
|  | GS55_2014 | 0.58 | 1.81 | 0.11 | 42.94 |  |  |  |  |  |  |  |  |  |  |
|  | GS55_meta |  |  | 0.12 | 35.61 |  |  |  |  |  |  |  |  |  |  |
| *QFt.niab-7B.04* | BLUE_2012 |  |  | 0.95 | 36.77 |  |  |  |  |  |  |  |  |  |  |
| *QFt.niab-7B.05* | GS_M2012 | 0.53 | 0.70 |  |  |  |  |  |  |  |  |  |  | 0.54 | NA |
|  | GS39_2014 |  |  | 0.35 | 49.17 |  |  | 0.33 | 49.36 |  |  |  |  |  |  |
|  | GS61_2014 |  | 2.18 |  |  |  |  |  |  |  |  |  |  | 0.66 | NA |

**Supplementary Table S9.** List of all detected quantitative trait loci (QTL) per trial year, where ‘X’ indicates trial years in which the QTL was detected in at least one analysis in that trialling year or across the meta-analyses.

|  | **Trialling year** | | | | | | |
| --- | --- | --- | --- | --- | --- | --- | --- |
| **‘Major’ QTL** | **2011** | **2012** | **2013** | **2014** | **2015** | **2016** | **Meta** |
| *QFt.niab-1B.05* | X | X | X | X | X | X | X |
| *QFt.niab-1D.03* | X | X | X | X | X | X | X |
| *QFt.niab-2D.01* | X | X | X | X | X | X | X |
| *QFt.niab-3A.01* | X | X | X | X | X | X | X |
| *QFt.niab-4A.03* | X | X | X | X | X | X | X |
| *QFt.niab-4B.01* | X | X | X | X | X | X | X |
| *QFt.niab-4D.01* |  | X | X | X |  | X | X |
| *QFt.niab-6A.03* | X | X | X | X | X | X | X |
| *QFt.niab-6D.01* | X | X | X | X | X | X | X |
| *QFt.niab-7B.01* | X | X | X | X | X |  | X |
| **‘Minor’ QTL** |  |  |  |  |  |  |  |
| *QFt.niab-1A.01* |  |  | X | X |  |  | X |
| *QFt.niab-1A.02* |  | X |  | X |  |  | X |
| *QFt.niab-1A.03* |  |  | X |  |  |  |  |
| *QFt.niab-1A.04* |  |  | X |  |  |  |  |
| *QFt.niab-1B.01* |  |  | X |  | X |  | X |
| *QFt.niab-1B.02* |  | X |  | X | X |  | X |
| *QFt.niab-1B.03* |  | X |  | X | X |  | X |
| *QFt.niab-1B.04* |  | X |  | X | X | X | X |
| *QFt.niab-1D.01* | X |  |  |  |  |  |  |
| *QFt.niab-1D.02* |  |  | X |  |  |  |  |
| *QFt.niab-2A.01* |  |  |  |  | X | X |  |
| *QFt.niab-2A.02* |  | X |  |  |  |  |  |
| *QFt.niab-2A.03* |  | X |  |  |  |  |  |
| *QFt.niab-2A.04* |  |  |  | X |  |  | X |
| *QFt.niab-2B.01* |  | X | X |  |  |  |  |
| *QFt.niab-2B.02* | X |  |  |  | X |  |  |
| *QFt.niab-2B.03* |  |  |  |  | X |  |  |
| *QFt.niab-2D.02* |  | X | X |  |  |  |  |
| *QFt.niab-4A.01* |  | X |  | X | X | X | X |
| *QFt.niab-4A.02* | X | X |  |  |  | X |  |
| *QFt.niab-4D.02* |  |  | X |  |  |  | X |
| *QFt.niab-5A.01* |  |  | X |  |  |  |  |
| *QFt.niab-5A.02* |  | X | X |  | X |  |  |
| *QFt.niab-5A.03* |  | X | X | X | X | X | X |
| *QFt.niab-5A.04* |  | X |  | X |  | X | X |
| *QFt.niab-5A.05* |  |  |  | X | X |  | X |
| *QFt.niab-5B.01* |  |  | X |  |  |  |  |
| *QFt.niab-5B.02* | X |  |  | X |  |  | X |
| *QFt.niab-5B.03* | X | X | X | X | X | X | X |
| *QFt.niab-5B.04* |  |  |  |  | X |  |  |
| *QFt.niab-5B.05* |  | X |  |  |  |  | X |
| *QFt.niab-5D.01* |  | X |  |  |  |  | X |
| *QFt.niab-5D.02* | X |  |  | X | X |  | X |
| *QFt.niab-5D.03* |  | X |  | X |  |  |  |
| *QFt.niab-6A.01* |  |  |  | X |  |  |  |
| *QFt.niab-6A.02* |  | X |  |  |  |  |  |
| *QFt.niab-6B.01* |  |  |  | X |  |  | X |
| *QFt.niab-6B.02* |  |  | X |  | X |  |  |
| *QFt.niab-7A.01* |  | X |  |  |  |  |  |
| *QFt.niab-7A.02* |  |  | X |  |  | X |  |
| *QFt.niab-7A.03* |  |  | X |  |  |  |  |
| *QFt.niab-7A.04* |  |  |  | X |  |  | X |
| *QFt.niab-7A.05* |  |  |  |  | X |  |  |
| *QFt.niab-7B.02* |  |  |  |  | X |  |  |
| *QFt.niab-7B.03* |  | X | X | X |  |  | X |
| *QFt.niab-7B.04* |  | X |  |  |  |  |  |
| *QFt.niab-7B.05* |  | X |  | X |  |  |  |

**Supplementary Table S10.** The 31 quantitative trait loci (QTL) identified as interacting with the major photoperiod response locus, *Ppd-D1*. Of these, 11 were in the same physical interval as the main effect QTL (Fixed QTL). Fixed QTL classified in this manuscript as ‘major’ QTL are highlighted in bold. SNPs physical map positions are relative to the wheat reference genome of Chinese Spring (RefSeq v1.0, IWGSC *et al.,* 2018). Chr = chromosome. Max_sign = the maximum significance value. Max_sign denotes the maximum significance above the threshold calculated as (-log_10_(*p*-value)-threshold). Unk = sequence not located on chromosome scaffold in the wheat reference genome.

| **Chr.** | **Interaction QTL** | **Fixed QTL** | **SNP** | **Chr. (Mbp position)** | **Method** | **Trait** | **Max_sign** |
| --- | --- | --- | --- | --- | --- | --- | --- |
| 1A | *QFt.Ppd_interaction.1A.01* | *QFt.niab-1A.04* | BS00032825_51 | 1A (557.1) | IBD_PPD | GS39_2014 | 0.246 |
|  | *QFt.Ppd_interaction.1A.02* |  | IACX6022 | 1A (589.1) | SNP_PPD | GS39_2014 |  |
| 1B | *QFt.Ppd_interaction.1B.01* |  | BS00074911_51 | 1B (626.2) | IBD_PPD | GS_M2012 | 0.067 |
|  | *QFt.Ppd_interaction.1B.02* |  | BS00003633_51 | 1B (633.1) | SNP_PPD | GS39_2014 | 0.656 |
|  |  |  |  |  |  | GS61_2014 |  |
|  | *QFt.Ppd_interaction.1B.03* |  | BS00110435_51a | 1B (636.7) | SNP_PPD, IBD_PPD | GS39_2014 | 0.248 |
|  |  |  |  |  |  | GS61_2014 |  |
| 1D | *QFt.Ppd_interaction.1D.01* | ***QFt.niab-1D.03*** | RAC875_c41914_613 | 1D (485.9) | SNP_PPD, IBD_PPD | GS39_2014 | 0.948 |
|  |  |  |  |  |  | GS39_2015 |  |
|  |  |  |  |  |  | GS39_meta |  |
|  |  |  |  |  |  | GS55_2015 |  |
|  |  |  |  |  |  | GS55_meta |  |
|  |  |  |  |  |  | GS61_meta |  |
| 2A | *QFt.Ppd_interaction.2A.01* |  | Excalibur_c39493_251 | 2A (3.9) | SNP_PPD, IBD_PPD | GS39_2015 | 1.458 |
|  |  |  |  |  |  | GS39_2015 |  |
|  |  |  |  |  |  | GS55_2015 |  |
|  | *QFt.Ppd_interaction.2A.02* |  | BS00055665_51 | 2A (691.8) | SNP_PPD | GS39_2015 | 0.464 |
| 2B | *QFt.Ppd_interaction.2B.01* | *QFt.niab-2B.02* | GENE_1119_104 | 2B (786.0) | SNP_PPD, IBD_PPD | GS_M2012 | 1.734 |
| 2D | *QFt.Ppd_interaction.2D.01* |  | BS00111139_51 | 2D (10.8) | SNP_PPD | GS61_2015 | 0.768 |
|  |  |  |  |  |  | GS61_meta |  |
|  | *QFt.Ppd_interaction.2D.02* | ***QFt.niab-2D.01*** | GENE_0137_147 | 2D (29.2) | SNP_PPD, IBD_PPD | GS39_2014 | 1.66 |
|  |  |  |  |  |  | GS39_2015 |  |
|  |  |  |  |  |  | GS39_meta |  |
|  |  |  |  |  |  | GS55_2015 |  |
|  |  |  |  |  |  | GS61_2014 |  |
|  | *QFt.Ppd_interaction.2D.03* | *QFt.niab-2D.02* | BobWhite_c6770_617 | 2D (420.5) | SNP_PPD | GS39_2014 | 0.507 |
| 3B | *QFt.Ppd_interaction.3B.01* |  | wsnp_Ex_c3005_5548573 | 3B (26.7) | SNP_PPD | GS39_2014 | 0.814 |
|  |  |  |  |  |  | GS39_meta |  |
|  | *QFt.Ppd_interaction.3B.02* |  | GENE_1455_114 | 3B (556.9) | SNP_PPD, IBD_PPD | GS_J2012 | 2.426 |
|  |  |  |  |  |  | GS_M2012 |  |
|  | *QFt.Ppd_interaction.3B.03* |  | BS00009549_51 | 3B (705.8) | SNP_PPD | GS_M2012 | 0.415 |
|  | *QFt.Ppd_interaction.3B.04* |  | BS00071042_51 | 3B (796.3) | SNP_PPD | GS_M2012 | 0.68 |
| 3D | *QFt.Ppd_interaction.3D.01* |  | RAC875_c7727_1458 | 3D (3.3) | SNP_PPD, IBD_PPD | GS_M2012 | 2.177 |
|  |  |  |  |  |  | GS61_2015 |  |
|  |  |  |  |  |  | GS61_meta |  |
| 4A | *QFt.Ppd_interaction.4A.01* |  | BobWhite_rep_c66057_98 | 4A (38.4) | IBD_PPD | GS61_2016 | 0.728 |
| 4B | *QFt.Ppd_interaction.4B.01* | ***QFt.niab-4B.01*** | Ex_c45493_761 | 4B (643.5) | SNP_PPD | GS61_2014 | 0.372 |
| 4D | *QFt.Ppd_interaction.4D.01* |  | D_GDRF1KQ02H66WD_341 | Unk | IBD_PPD | GS_M2012 | 0.559 |
| 5A | *QFt.Ppd_interaction.5A.01* | *QFt.niab-5A.04* | BobWhite_c17045_186 | 5A (666.7) | SNP_PPD, IBD_PPD | BLUE_2012 | 1.665 |
|  |  |  |  |  |  | GS39_2016 |  |
|  |  |  |  |  |  | GS55_2014 |  |
|  |  |  |  |  |  | GS61_2014 |  |
|  |  |  |  |  |  | GS61_2016 |  |
|  |  |  |  |  |  | GS61_meta |  |
| 5B | *QFt.Ppd_interaction.5B.01* |  | Excalibur_rep_c68199_123 | 5B (317.3) |  | GS55_2013 | 0.737 |
| 6A | *QFt.Ppd_interaction.6A.01* | *QFt.niab-6A.01* | Tdurum_contig55193_296 | 6A (6.6) | SNP_PPD, IBD_PPD | GS_M2012 | 0.836 |
|  |  |  |  |  |  | GS39_2014 |  |
|  |  |  |  |  |  | GS39_2015 |  |
|  |  |  |  |  |  | GS39_meta |  |
|  | *QFt.Ppd_interaction.6A.02* |  | IAAV5088 | 6A (600.7) | SNP_PPD | GS_M2012 | 0.325 |
|  | *QFt.Ppd_interaction.6A.03* |  | BS00082460_51 | 6A (615.6) | SNP_PPD, IBD_PPD | BLUE_2011 | 3.600 |
|  |  |  |  |  |  | BLUE_2012 |  |
|  |  |  |  |  |  | GS_M2012 |  |
| 6B | *QFt.Ppd_interaction.6B.01* |  | BS00077896_51 | 6B (18.6) | IBD_PPD | GS39_2015 | 0.163 |
| 6D | *QFt.Ppd_interaction.6D.01* | *QFt.niab-6D.01* | CAP7_c1208_150 | 6D (2.3) | SNP_PPD | GS39_2015 | 0.607 |
|  |  |  |  |  |  | GS61_2014 |  |
|  | *QFt.Ppd_interaction.6D.02* | ***QFt.niab-6D.01*** | BobWhite_c13504_378 | 6D (460.6) | IBD_PPD | GS_M2012 | 0.189 |
| 7A | *QFt.Ppd_interaction.7A.01* |  | wsnp_Ex_c27898_37058842 | 7A (680.0) | SNP_PPD | GS_M2012 | 1.136 |
| 7B | *QFt.Ppd_interaction.7B.01* | ***QFt.niab-7B.01*** | wsnp_Ex_c46274_51831129 | 7B (42.0) | SNP_PPD, IBD_PPD | GS_M2012 | 1.901 |
|  | *QFt.Ppd_interaction.7B.02* | *QFt.niab-7B.05* | IACX5786 | 7B (742.5) | SNP_PPD, IBD_PPD | GS_M2012 | 0.854 |

**Supplementary Table S11.** Quantitative trait loci (QTL) detected in the HOH_2015 trial in Germany, including the corresponding QTL name in the main analyses conducted in the UK trials (those identified in the main analyses as ‘major’ QTL are highlighted in bold; *n.d.* denotes QTL not detected in the main analysis), the maximum significance value (Max_sign) and the genetic analysis method the QTL was detected with (SNP, SNP_PPD, IBD, IBD_PPD). Max_sign denotes the maximum significance above the threshold calculated as (-log_10_(*p*-value)-threshold).

| **Chr** | **QTL name** | **Max_sign** | **SNP** | **SNP_PPD** | **IBD** | **IBD_PPD** |
| --- | --- | --- | --- | --- | --- | --- |
| 1A | *QFt.niab-1A.01* | 0.81 | X | X | X | X |
| 1B | ***QFt.niab-1B.05*** | 0.75 |  | X |  | X |
| 1D | ***QFt.niab-1D.03*** | 2.23 | X | X | X | X |
| 2D | ***QFt.niab-2D.01*** | 12.68 | X |  | X |  |
| 3A | ***QFt.niab-3A.0****1* | 4.17 | X | X | X | X |
| 3B | *n.d.* | 0.18 |  |  |  | X |
| 4A | ***QFt.niab-4A.03*** | 4.52 | X | X | X | X |
| 5A | *QFt.niab-5A.03* | 0.17 |  | X |  |  |
| 5A | *QFt.niab-5A.05* | 0.43 |  |  | X | X |
| 6A | *Q****Ft.niab-6A.03*** | 0.66 | X |  |  |  |
| 6B | *QFt.niab-6B.01* | 0.51 |  |  |  | X |
| 6B | *n.d.* | 0.27 |  |  | X | X |

**Supplementary Table S12.** See separate Excel file.

**Supplementary Table S13.** Flowering time scores for the near isogenic line (NIL) for ‘minor’ QTL *QFt.niab-5A.03* phenotyped in 1x1 m^2^ ‘nursery’ plots under field conditions in 2022 and 2023. The NIL was created via exploitation of residual heterozygosity in MAGIC recombinant inbred line (RIL) MEL_084_2. This RIL segregated for alleles from the founders ‘Hereward’ and ‘Soissons’ at the locus, predicted to confer later and earlier flowering, respectively. Analysis of flowering time in the NIL pair confirmed the predicted allelic effects, finding the NIL lines with the ‘Hereward’ allele (genotype call B:B) to flower later than the NIL lines with the ‘Soissons’ allele. GS55 = Zadoks growth stage 55 (half of ear emerged above flag leaf ligule), measured as days to reach GS55 from the 1^st^ May. **^†^**Indicates the individual plant which was genotyped using a KASP marker at the target QTL locus to determine genotype as A:A, A:B or B:B. Self-pollinated seed from these plants were subsequently harvested and used to sow the 1×1 m^2^ nursery plots assessed here for GS55 in 2022, with an individual plant from several of these lines used as seed for the 2023 plots. **^‡^** A two-sample Welch’s t-test of the replicated plots for the early (Soissons) and late (Hereward) alleles undertaken in the 2023 field trial showed a significant effect associated with allele (*t* = -3.29, *p*-value = 0.02).

| **Line^†^** | **SNP genotype^†^** | **Founder allele** | **2022 nursery plot number** | **Days to GS55** | **2023 plot numbers** | **Days to GS55^‡^** |
| --- | --- | --- | --- | --- | --- | --- |
| MEL_084_2_2_12 | B:B | Hereward | 35 | 32 | 27_3, 27_8, 27_12 | 43, 44, 41 |
| MEL_084_2_2_13 | B:B | Hereward | 36 | 32 | 27_5, 27_6, 27_9 | 44, 44, 44 |
| MEL_084_2_2_04 | A:A | Soissons | 32 | 26 | 27_2, 27_10 | 27, 27 |
| MEL_084_2_2_19 | A:A | Soissons | 37 | 27 | 27_7, 27_13 | 41, 36 |
| MEL_084_2_2_20 | A:A | Soissons | 38 | 27 | 27_4, 27_11 | 41, 36 |
| MEL_084_2_2_23 | A:A | Soissons | 40 | 28 | NA | NA |
| MEL_084_2_2_01 | A:B | Hereward:Soissons | 31 | 31 | NA | NA |
| MEL_084_2_2_05 | A:B | Hereward:Soissons | 33 | 30 | NA | NA |
| MEL_084_2_2_09 | A:B | Hereward:Soissons | 34 | 29 | NA | NA |
| MEL_084_2_2_22 | A:B | Hereward:Soissons | 39 | 30 | NA | NA |
